# Decoupling Neuroprotection from MAO Inhibition via Indane-to-Azaindane Bioisosteric Replacement of Rasagiline

**DOI:** 10.1021/acsmedchemlett.6c00407

**Published:** 2026-09-12

**Authors:** Ivo E. Sampaio-Dias, Sara C. Silva-Reis, Xavier C. Correia, Hugo F. Costa-Almeida, Maria B. Igreja-Cardoso, Gabriel M. Azevedo, Vera M. Costa, Damijan Knez, Stanislav Gobec, José E. Rodríguez-Borges

**Affiliations:** † LAQV/REQUIMTE, Department of Chemistry and Biochemistry, Faculty of Sciences, University of Porto, Rua do Campo Alegre s/n, 4169-007 Porto, Portugal; ‡ UCIBIO - Applied Molecular Biosciences Unit, Laboratory of Toxicology, Department of Biological Sciences, Faculty of Pharmacy, University of Porto, 4050-313 Porto, Portugal; § Associate Laboratory i4HB - Institute for Health and Bioeconomy, Faculty of Pharmacy, University of Porto, 4050-313 Porto, Portugal; ∥ 37663 University of Ljubljana, Faculty of Pharmacy, Aškerčeva 7, 1000 Ljubljana, Slovenia

**Keywords:** Azaindane, Parkinson’s Disease, Rasagiline, Regioselective Oxidation

## Abstract

Rasagiline is a monoamine oxidase B (MAO-B) inhibitor with neuroprotective effects, whose dependence on MAO-B inhibition remains unclear. Here, indane-to-azaindane rasagiline isosteres (*rac*-**2a** and *rac*-**2b**) were developed to investigate whether neuroprotection can be decoupled from MAO-B inhibition. Direct regioselective oxidation of 4-azaindane catalyzed by Mn­(OAc)_2_/^*t*^BuOOH afforded the key 4-azaindan-1-one intermediate (**6a**). In paraquat-insulted dopaminergic differentiated SH-SY5Y cells, both analogs showed neuroprotection at 10 and 100 μM, with *rac*-**2b** outperforming rasagiline. The azaindane analogs displayed markedly reduced MAO-B inhibition and no time-dependent inhibition. These findings demonstrate that rasagiline neuroprotection can be dissociated from MAO-B inhibition, supporting azaindane bioisosterism for exploring MAO-independent neuroprotection.

## Graphical abstract

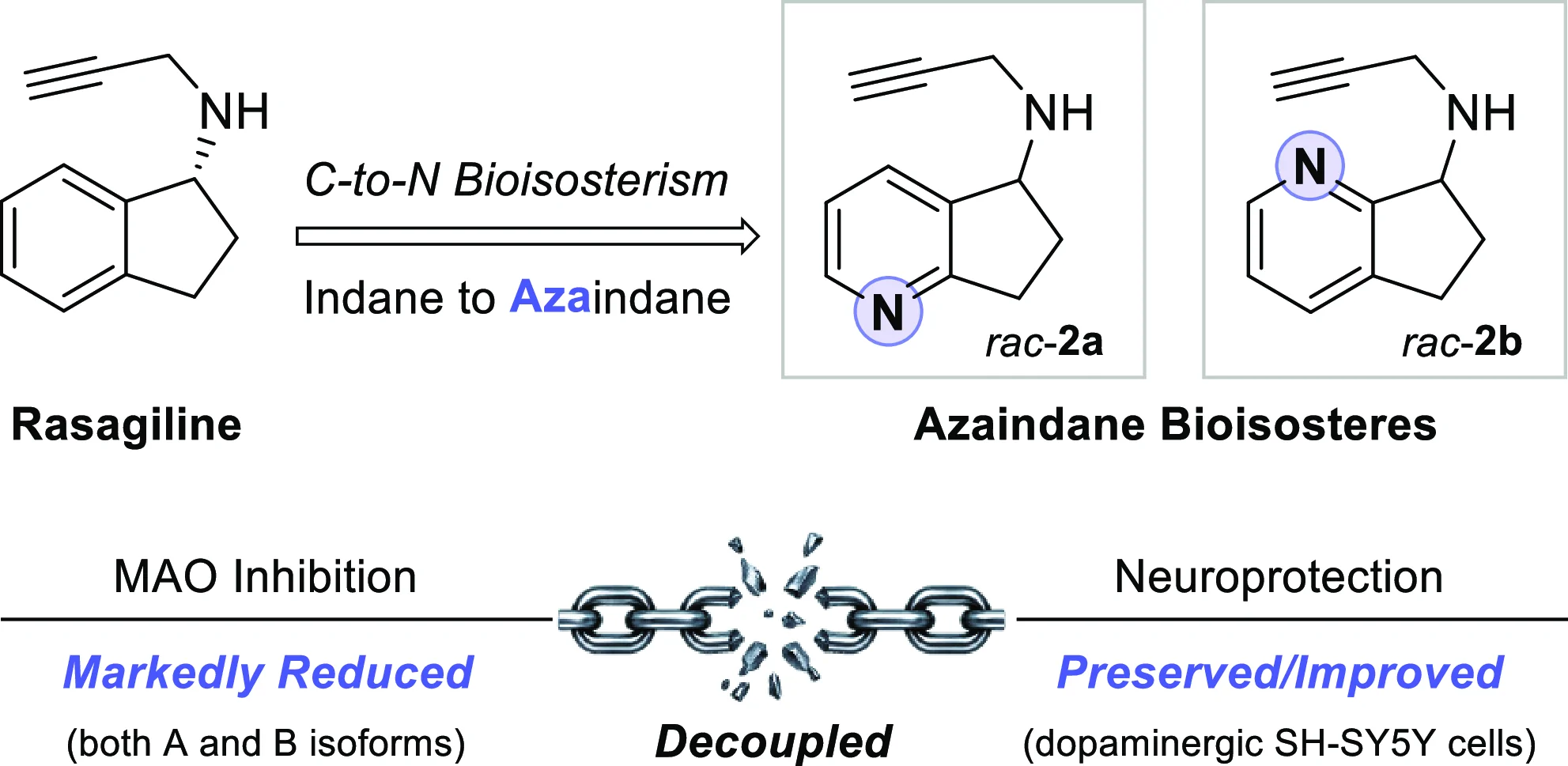

Parkinson’s disease (PD) is a progressive neurodegenerative disorder characterized by the selective and progressive loss of dopaminergic neurons in the *substantia nigra* and the consequent depletion of striatal dopamine levels.^1^ A central feature of PD pathogenesis is oxidative stress, arising from mitochondrial dysfunction, impaired redox homeostasis, and dopamine metabolism.^2^

Monoamine oxidase B (MAO-B), predominantly expressed in glial cells, plays a central role in this process by catalyzing the oxidative deamination of monoamines, generating hydrogen peroxide and other reactive byproducts.^3,4^ These species contribute to oxidative damage, lipid peroxidation, and neuronal death,^4^ and are further implicated in the bioactivation of neurotoxins such as MPTP to MPP^+^.^5^ Accordingly, MAO-B inhibition has long been considered a rational strategy to reduce oxidative stress and confer neuroprotection, as well as increase dopamine levels.^5^

(*R*)-*N*-Propargyl-1-aminoindane, commonly known as rasagiline (**1**, Figure 1), is the active pharmaceutical ingredient of Azilect, which is marketed as a mesylate salt.^6–8^ It is widely used to treat PD, either as monotherapy or in combination with levodopa and carbidopa to control motor symptoms.^9–16^ Chemically, rasagiline consists of a secondary cyclic benzylamine (1-aminoindane) scaffold bearing an *N*-propargyl substituent.^17^ Pharmacologically, it acts as a selective, irreversible MAO-B inhibitor by covalently modifying the flavin cofactor.^17^ Its activity is stereochemically dependent, with the (*R*)-enantiomer responsible for potent MAO-B inhibition, whereas the (*S*)-enantiomer (TVP 1022) displays markedly reduced inhibitory activity, with a 10^4^-fold increase in the half-maximal inhibitory concentration (IC_50_ value).^6,7^

**Figure 1:**
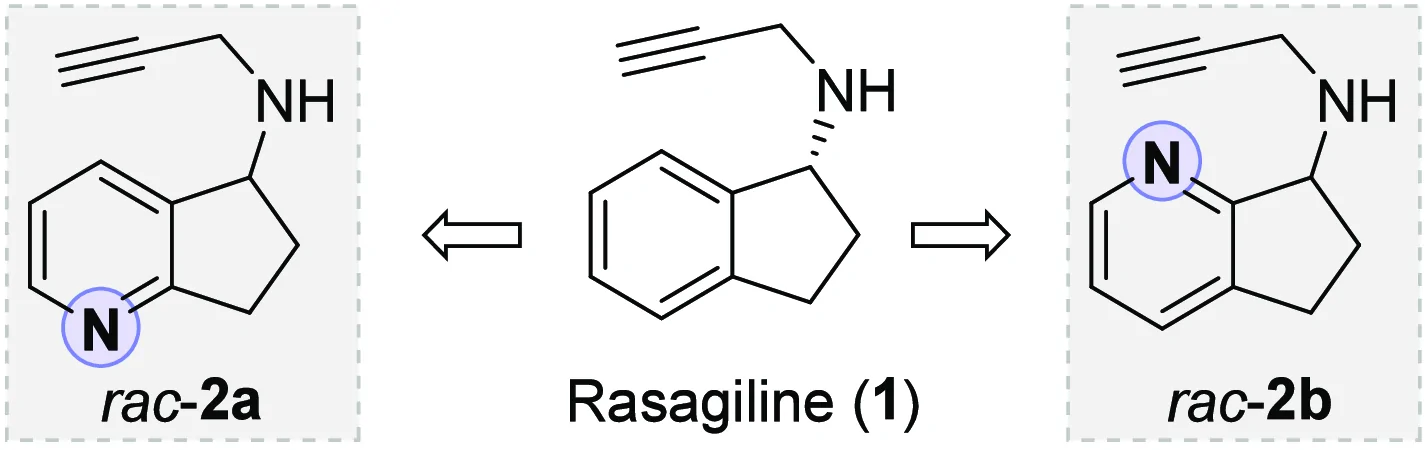
Structures of rasagiline (**1**) and its azaindane-based isosteres *rac*-**2a** and *rac*-**2b**.

Beyond its dopaminergic effects, rasagiline has consistently been reported to exert neuroprotective activity across multiple experimental models, including protection against oxidative stress, mitochondrial dysfunction, and apoptotic signaling pathways.^18–22^ However, growing evidence suggests that these effects are not solely attributable to MAO-B inhibition.^23^ Notably, both enantiomers exhibit comparable neuroprotective effects, indicating that additional mechanisms may contribute to their activity,^24,25^ consistent with studies on TVP 1022, which retains significant neuroprotective activity despite weak MAO-B inhibition.^23,26^

These effects have been associated with the *N*-propargyl pharmacophore and have been implicated in mitochondrial stabilization and antiapoptotic signaling, including modulation of Bcl-2 family proteins and attenuation of mitochondrial permeability transition pore opening.^19,27,28^ Moreover, the principal metabolite of rasagiline, (*R*)-1-aminoindane, displays antioxidant and neuroprotective effects, potentially reinforcing the overall protective profile attributed to rasagiline.^14^ These findings suggest that rasagiline neuroprotection may arise from MAO-independent mechanisms,^28^ although the relative contribution of each pathway remains unclear.

This mechanistic ambiguity complicates the interpretation of rasagiline pharmacology and raises a fundamental question: to what extent is its neuroprotective activity intrinsically dependent on MAO-B inhibition? Addressing this question requires molecular tools capable of uncoupling these pharmacological properties. In this context, bioisosteric molecular editing represents a particularly attractive medicinal chemistry strategy, as it enables the selective modulation of target engagement while preserving the broader physicochemical and pharmacological profile of the parent scaffold. Accordingly, the design of rasagiline analogs that selectively abolish MAO-B inhibition while retaining neuroprotective activity offers a unique opportunity to define the molecular determinants underlying each function.^9–16,29–33^ Beyond providing mechanistic insight into rasagiline pharmacology, such compounds could lay the foundation for a new generation of neuroprotective agents devoid of irreversible MAO inhibition and its associated mechanism-based liabilities, including tyramine-mediated dietary interactions and other MAO-related adverse effects.^34^

Guided by this rationale, we focused on the 1-aminoindane bicyclic scaffold of rasagiline, which plays a critical role in MAO-B recognition by inducing the “open” conformation of the gating residue Ile199 (PDB: 1S2Q).^17^ We hypothesized that replacing this core with an azaindane bioisostere would perturb key enzyme-ligand interactions, as previously observed for rasagiline-related inhibitors,^33^ thereby attenuating or abrogating MAO-B inhibition while potentially preserving structural determinants responsible for MAO-independent neuroprotective activity.

Our research group has been exploring 4-azaindane (6,7-dihydro-5*H*-cyclopenta­[*b*]­pyridine, CPP) as an indane surrogate (Figure 1).^16,35,36^ The incorporation of a nitrogen atom within the aromatic framework is expected to alter the electronic properties of the scaffold and potentially modulate its interactions within the MAO-B active site.^16^ Despite the increased polarity introduced by the ring nitrogen, the resulting physicochemical profile remains compatible with blood-brain barrier permeation, as supported by *in silico* analysis (Supporting Information). Azaindane scaffolds are also found in diverse bioactive molecules, including cephalosporins such as cefpirome sulfate, a fourth-generation cephalosporin,^37^ or as endothelin receptor antagonists, which are used in renal disease, hypertension, and congestive heart failure.^38^ Other studies suggest that azaindanes might be used as antifungals^39^ and phosphatase CDC25 inhibitors.^40,41^

In a previous study, we reported the synthesis of 1-(propargylamino)-7-azaindane (*rac*-**2b**, Figure 1).^35,36^ Building on that work, we investigate the effect of azaindane regioisomers (*rac*-**2a** vs *rac*-**2b**) on neuroprotective activity and MAO inhibition, compared with rasagiline. While protocols for the preparation of *rac*-**2b** are well established based on the key Boekelheide rearrangement,^16,35,36,42^ synthesis routes for the preparation of *rac*-**2a** are not.

Herein, we report the design and synthesis of indane-to-azaindane rasagiline isosteres through regioselective functionalization of the 4-azaindane scaffold. We describe an effective and straightforward synthetic route enabling the regioselective preparation of *rac*-**2a** via direct functionalization at the 1-position of the 4-azaindane system, alongside its previously reported structural isomer (*rac*-**2b**). Their neuroprotective effects were evaluated in a dopaminergic SH-SY5Y cell model of paraquat (PQ)-induced oxidative stress, while MAO inhibitory activity was assessed using a cell-free enzymatic assay. This approach enabled direct assessment of structure–activity relationships and the disentanglement of neuroprotection from MAO inhibition for this chemotype.

## Organic Chemistry

The synthesis of *rac*-**2a** started with the preparation of ketone **6a** (Scheme 1), a costly intermediate commonly employed in the synthesis of antipsychotic agents.^43,44^ Our initial approach toward ketone **6a** involved a superacid-promoted intramolecular cyclization of ester **5**, under conditions typically used for indanone formation.^45^ Starting from aldehyde **3** (Scheme 1), conjugated ester **4** was obtained via a Wittig reaction using the stabilized ylide (carbethoxymethylene)­triphenylphosphorane in 82% yield, with complete *E*-selectivity (^1^H NMR, *J* = 15.7 Hz). Subsequent hydrogenation of **4** over 10% Pd/C afforded saturated ester **5** in excellent yield (99%). However, attempted cyclization of **5** to azaindanone **6a** using triflic acid proved unsuccessful, with complete recovery of starting material.

**Scheme 1:**
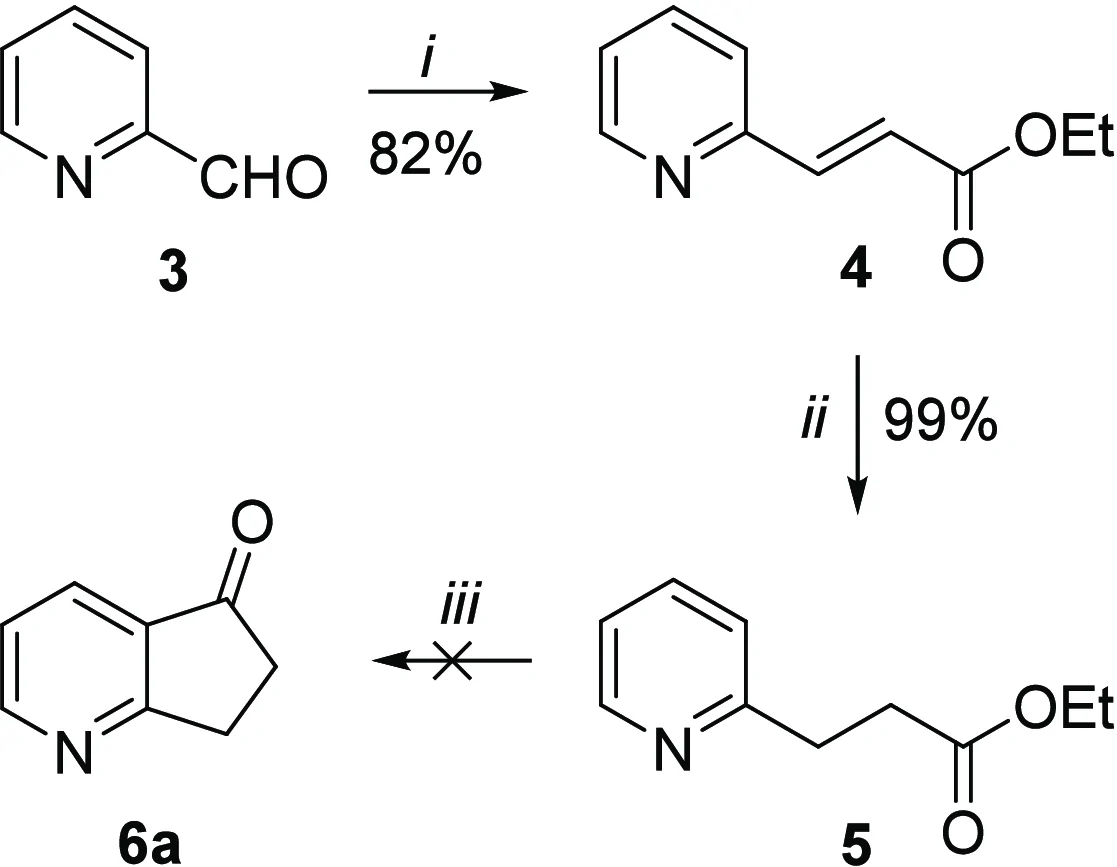
Attempted Synthesis of 4-Azaindan-1-one (**6a**) *via* Cyclization of Ester **5** ^a^

a Reagents and conditions: (*i*) (carbethoxymethylene)­triphenylphosphorane, anhydrous CH_2_Cl_2_, 25 °C, 24 h; (*ii*) H_2_ (3.45 bar), Pd/C, EtOAc, 25 °C, 1 h; (*iii*) triflic acid (neat), 25 °C, 24 h.

To circumvent this limitation, we investigated the direct regioselective oxidation of CPP (Scheme 2) to ketone **6a**, which is known to be challenging.^43,44^ Oxidation of CPP using CrO_3_ in a mixture of acetic and sulfuric acids afforded **6a** in low yield (10%).^43^ More recently, electrochemical^46,47^ and photochemical approaches have been reported,^48^ providing moderate yields (41–57%);^46–48^ however, these methods require specialized equipment and setups.^46–48^

**Scheme 2:**
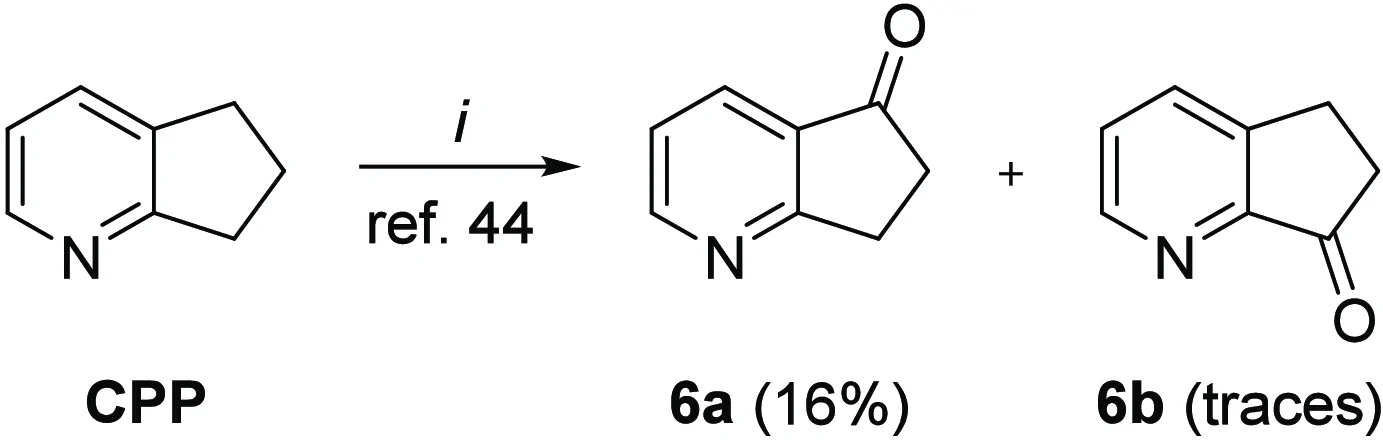
Synthesis of Ketone **6a** Following the Protocol of Ren and Co-workers^44^ ^,^ ^a^

a Reagents and conditions: (*i*) Mn­(OTf)_2_, H_2_O, ^*t*^BuOOH (70% in H_2_O), 25 °C, 72 h.

Iron-catalyzed oxidative functionalization of CPP has also been described, delivering **6a** in 40% yield using 30% H_2_O_2_ as the oxidant, polyoxometalate (NH_4_)_3_[FeMo_6_O_18_(OH)_6_] as the catalyst, and ethyl 2-bromoacetate as an additive.^49^ In addition, a greener manganese-catalyzed oxidation protocol in water, employing *tert*-butyl hydroperoxide (^*t*^BuOOH) as the oxidant and manganese triflate (0.5 mol %) as the catalyst, was reported by Ren and co-workers (Scheme 2).^44^

Following this protocol (Scheme 2), ketone **6a** was successfully isolated, along with its positional regioisomer (ketone **6b**, Scheme 2) as a minor byproduct. The similar retention factors of **6a** and **6b** made their chromatographic separation particularly challenging, resulting in lower isolated yields of **6a** than those previously reported (maximum yield of 16% vs 88% and 68% reported at 0.5 and 25 mmol scales,^44^ respectively). Importantly, the low yields obtained do not necessarily reflect the intrinsic efficiency of the oxidation process.

Considering the poor solubility of CPP in water, nonaqueous solutions of ^*t*^BuOOH were evaluated for the regioselective oxidation of CPP. A 5.5 M solution of ^*t*^BuOOH in nonane was selected as oxidant. In addition, several manganese­(II) salts, including MnCl_2_, Mn­(OAc)_2_, and Mn­(OTf)_2_, were screened for the oxidation of CPP to ketone **6a** under controlled conditions. Among the catalysts tested, Mn­(OAc)_2_ provided the best results.

The optimal conditions employed 5 equiv of ^*t*^BuOOH and 5 mol % Mn­(OAc)_2_, with a reaction time of 48 h (Table S2). Under these conditions, chromatographic purification was significantly simplified due to the reduced number of side products observed by TLC, affording ketone **6a** in moderate yield (42%, Scheme 3). Notably, regioisomer **6b** was not detected by NMR analysis of the crude reaction mixture, indicating a highly regioselective oxidation. The observed regioselectivity may arise from the different coordinating properties of acetate and triflate, together with the low-temperature addition of the oxidant (−20 °C) and the improved solubility of CPP in the nonaqueous hexane/nonane medium.

**Scheme 3:**
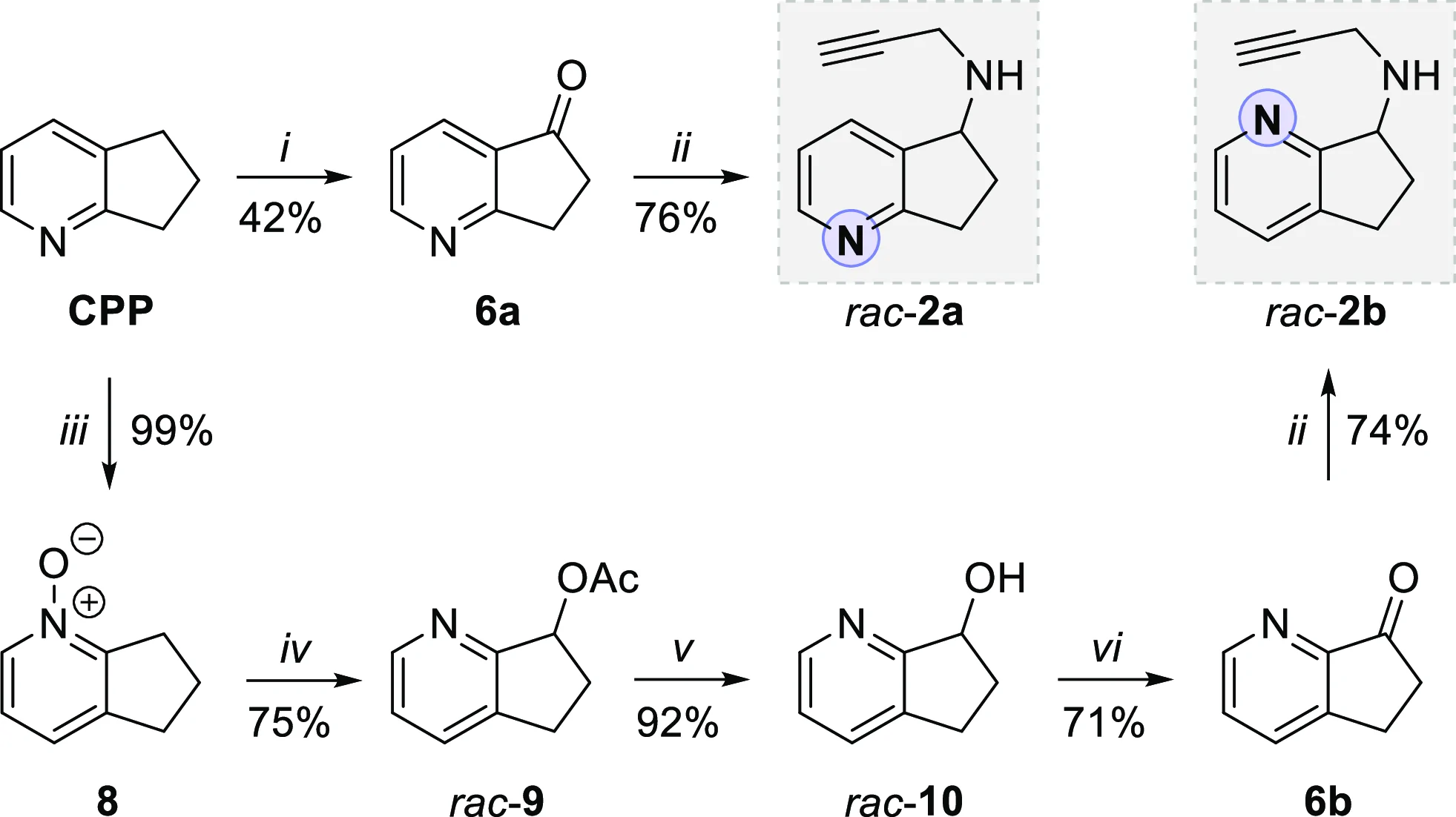
Synthesis of Rasagiline Isosteres *rac*-**2a** and *rac*-**2b** ^a^

a Reagents and conditions: (*i*) Mn­(OAc)_2_, dry hexane, ^*t*^BuOOH (5.5 M in nonane, added over 20 min at −20 °C), then 20 °C, 48 h; (*ii*) propargylamine, NaBH­(OAc)_3_, CH_2_Cl_2_, 25 °C, 24 h; (*iii*) *m*-CPBA, CH_2_Cl_2_, 25 °C, 2 h; (*iv*) Ac_2_O, 100 °C, 2 h; (*v*) KOH, EtOH, 25 °C, 1 h; (*vi*) 1. (CO_2_)­Cl_2_, CH_2_Cl_2_, DMSO, −78 °C; 2. Et_3_N, 20 h, −78 °C to room temperature.

With ketone **6a** in hand, rasagiline isostere *rac*-**2a** was readily obtained in good yield (76%, Scheme 3) via reductive amination with propargylamine using sodium triacetoxyborohydride, NaBH­(OAc)_3_, as the reducing agent.^36^

For the preparation of rasagiline isostere *rac*-**2b**, a regioselective synthetic route previously developed in our group was followed (Scheme 3).^35,36^ Briefly, CPP was oxidized using *meta*-chloroperbenzoic acid (*m*-CPBA) to the corresponding *N*-oxide (**8**) in near-quantitative yields (99%). Subsequent treatment with acetic anhydride (Ac_2_O) afforded ester *rac*-**9** via Boekelheide rearrangement (75%).^50,51^ Saponification of *rac*-**9** with KOH in ethanol (EtOH) delivered alcohol *rac*-**10** in 92% yield. Oxidation of alcohol *rac*-**10** under Swern conditions, namely oxalyl chloride and dimethyl sulfoxide (DMSO), followed by triethylamine (Et_3_N), furnished ketone **6b** in 71% yield. Finally, reductive amination with propargylamine afforded *rac*-**2b** in 74% yield (Scheme 3).

Since isosteres *rac*-**2a** and *rac*-**2b** were prepared as racemic mixtures, racemic rasagiline (*rac*-**1**) was also synthesized for comparison purposes via reductive amination of indan-1-one (**11**) with propargylamine, affording *rac*-**1** in 79% yield (Scheme 4).

**Scheme 4:**
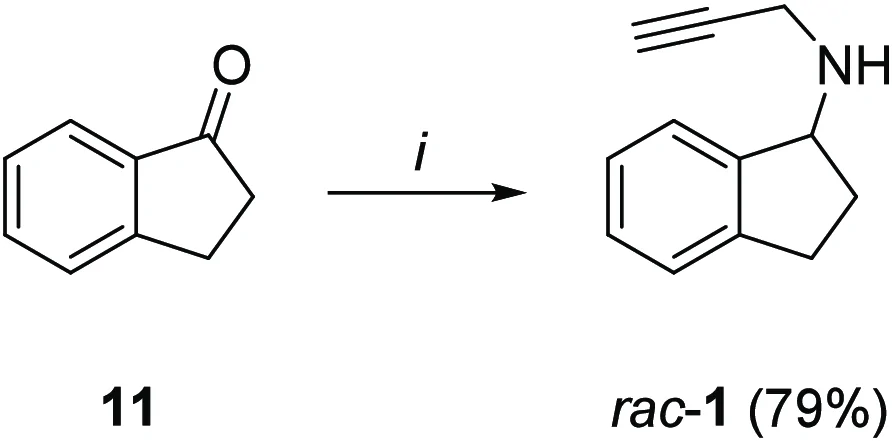
Synthesis of *rac* **-1** ^a^

a Reagents and conditions: (*i*) propargylamine, NaBH­(OAc)_3_, CH_2_Cl_2_.

With the target rasagiline isosteres *rac*-**2a** and *rac*-**2b** and reference analogs in hand, we next investigated their neurotoxicity and neuroprotective properties in a dopaminergic cell model relevant to PD.

## Neurotoxicity Assays

Differentiated SH-SY5Y cells were employed as the neuronal model. Differentiation into a dopaminergic-like phenotype was achieved following a well-established protocol routinely used in our group, involving sequential treatment with retinoic acid and phorbol 12-myristate 13-acetate, yielding a more mature, neuron-like phenotype. The acquisition of a dopaminergic phenotype was confirmed in previous work of the group using suitable neuronal markers,^52,53^ including increased expression of tyrosine hydroxylase and dopamine transporter.^52^ Oxidative stress was induced using PQ, a well-established neurotoxin, which was also used as a positive control for neurotoxicity. PQ is a widely used herbicide with well-documented neurotoxic properties that has been strongly associated with PD-like neurodegenerative alterations.^54^ Its toxicity in neuronal cells is primarily mediated by oxidative stress, mitochondrial dysfunction, and the selective vulnerability of dopaminergic neurons, key mechanisms implicated in PD pathogenesis.^55^ Accordingly, PQ-based *in vitro* models represent a relevant approach for evaluating the neuroprotective potential of candidate compounds against PD.

An initial screening was performed to assess the cytotoxicity of the rasagiline derivatives at 10 and 100 μM in differentiated SH-SY5Y cells using the MTT reduction assay (Figure 2A) and the neutral red (NR) uptake assay (Figure 2B). The selection of 10 μM was based on previous reports describing the biological activity of rasagiline in SH-SY5Y neuronal models under oxidative stress conditions.^56^

**Figure 2:**
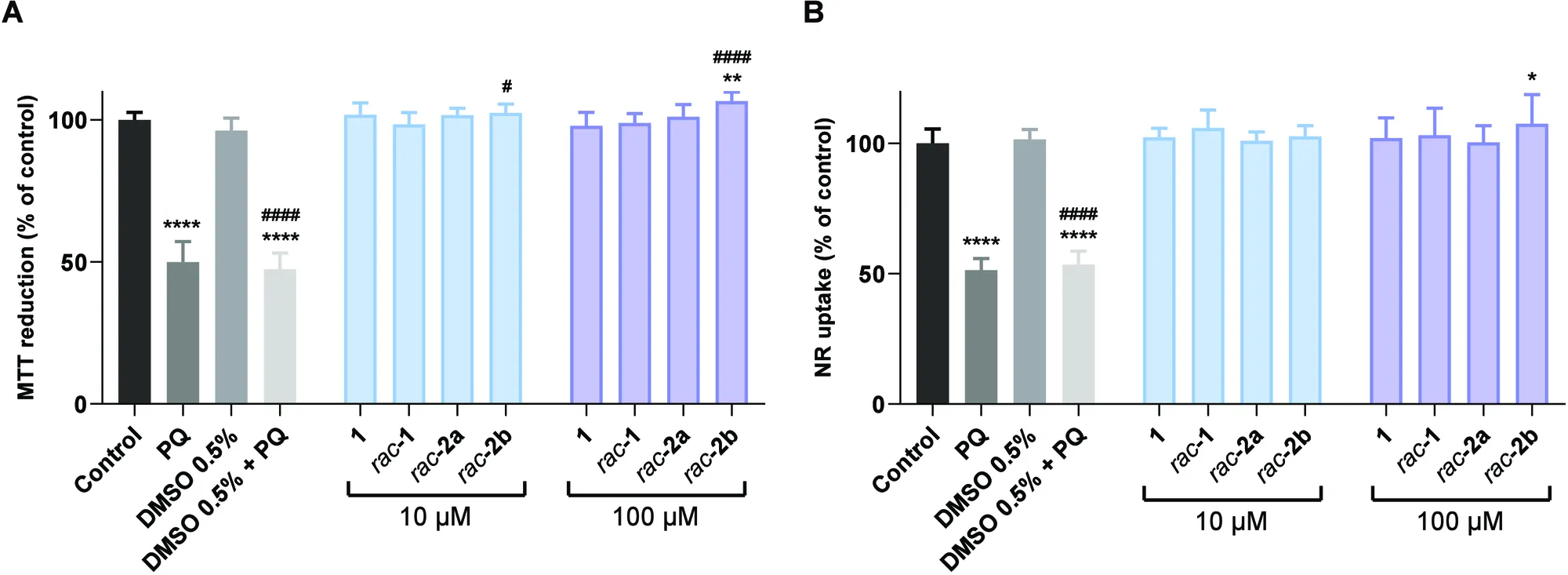
Cytotoxicity assays. MTT reduction (A) and NR uptake (B) assays performed in differentiated SH-SY5Y cells exposed to the selected rasagiline derivatives (10 and 100 μM, 48 h), PQ (300 μM), and the vehicle control for compound *rac*-**2b** (DMSO, 0.5% [v/v]). Data are expressed as a percentage of control (PBS) and presented as mean ± standard deviation. Results were obtained from at least three independent experiments performed in quadruplicate. Statistical analysis was performed using one-way ANOVA followed by Tukey *post hoc* test (*p < 0.05, **p < 0.01, and ****p < 0.0001 vs control; ^#^ *p* < 0.05, and ^####^ *p* < 0.0001 vs DMSO 0.5% condition).

Although those studies were conducted in nondifferentiated SH-SY5Y cells, they provide a relevant reference range for compound activity. The higher concentration (100 μM) was included as an upper exploratory dose to evaluate potential cytotoxic effects and to establish a safety margin for subsequent neuroprotection studies.

DMSO (0.5% [v/v] final concentration) was used as the vehicle for compound *rac*-**2b**, while the remaining compounds were dissolved in phosphate-buffered saline (PBS).

In both assays, PBS-treated cells served as the control group and were defined as 100% of control. Exposure to PQ resulted in significant cytotoxicity relative to control, as evidenced by decreased MTT reduction (49.91 ± 7.21%, ****p < 0.0001, Figure 2A) and NR uptake (51.32 ± 4.52%, ****p < 0.0001, Figure 2B), confirming the expected neurotoxic effect of PQ and validating the assays.

No significant changes were observed in the 0.5% DMSO-treated group compared with the control in both assays (MTT reduction and NR uptake), indicating that this vehicle itself did not influence cellular metabolic activity or lysosomal integrity (Figures 2A and 2B, respectively).

Among the tested compounds, no significant differences were observed relative to the respective PBS or 0.5% DMSO vehicles, except for *rac*-**2b**. In the MTT reduction assay, *rac*-**2b** showed a statistically significant increase relative to the 0.5% DMSO vehicle at 10 μM (102.42 ± 3.16%, ^#^ *p* < 0.05) and at 100 μM (106.56 ± 3.17%, ^####^ *p* < 0.0001), the latter being also significantly higher than the PBS vehicle (**p < 0.01). In the NR uptake assay, a significant increase was observed only at 100 μM relative to PBS (107.51 ± 11.20%, *p < 0.05). As the 0.5% DMSO caused no significant change, the observed increase cannot be attributed to the vehicle. Moreover, as the values only marginally exceeded 100%, the effect is unlikely to be biologically relevant.

Overall, these data demonstrate that compounds **1**, *rac*-**1**, *rac*-**2a**, and *rac*-**2b** do not exhibit cytotoxicity at concentrations up to 100 μM, in clear contrast to the pronounced cytotoxic effects induced by the neurotoxicant PQ, indicating that their half-maximal cytotoxic concentration (CC_50_) values exceed 100 μM.

## Neuroprotective Assays against PQ-Induced Toxicity

Following the initial cytotoxicity assessment, the selected compounds were evaluated for their potential neuroprotective effects against PQ-induced toxicity (300 μM) in differentiated SH-SY5Y neuroblastoma cells exhibiting a dopaminergic phenotype (Figure 3). The absence of compound-induced cytotoxicity under basal conditions (Figure 2) ensured that any effects observed in the presence of PQ could be attributed to neuroprotection rather than to intrinsic toxicity.

**Figure 3:**
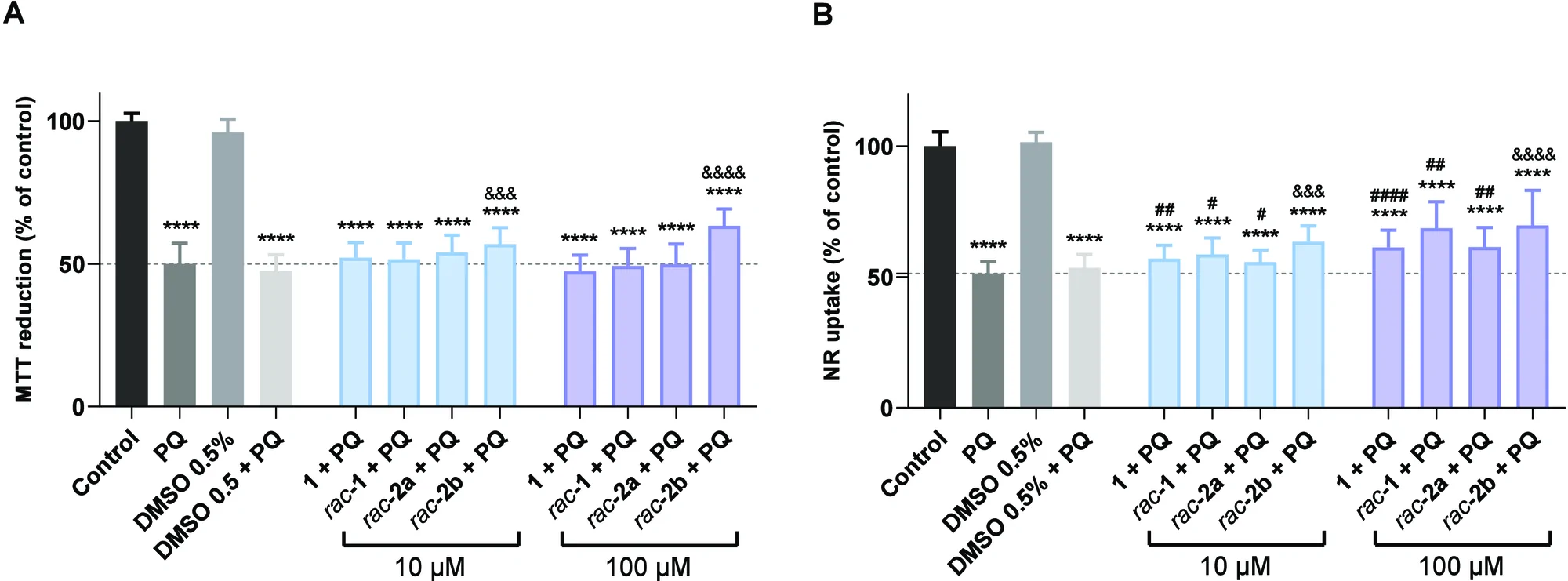
Neuroprotection assays. MTT reduction (A) and NR uptake (B) assays performed in differentiated SH-SY5Y cells coincubated with the selected rasagiline derivatives (10 and 100 μM, 48 h), including the vehicle for compound *rac*-**2b** (DMSO 0.5%), and PQ (300 μM). Data are expressed as a percentage of control (PBS) and are presented as mean ± standard deviation. Results were obtained from at least three independent experiments performed in quadruplicate. Statistical analysis was performed using one-way ANOVA followed by Tukey *post hoc* test (****p < 0.0001 vs control; ^#^ *p* < 0.05, ^##^ *p* < 0.01, and ^####^ *p* < 0.0001 vs PQ; ^&&&^ *p* < 0.001, ^&&&&^ *p* < 0.0001 vs DMSO 0.5% + PQ).

In the presence of PQ, *rac*-**2b** significantly conferred protection compared with the corresponding 0.5% DMSO + PQ vehicle control in both the MTT reduction and NR uptake assays (Figure 3). At 100 μM, *rac*-**2b** increased MTT reduction from 47.48 ± 5.64% to 63.34 ± 5.85% (^&&&&^ *p* < 0.0001) and NR uptake from 53.60 ± 5.10% to 69.70 ± 13.41% (^&&&&^ *p* < 0.0001), representing an approximately 16% improvement in both assays. Similar, although less pronounced, effects were observed at 10 μM, with values of 56.85 ± 5.82% (^&&&^ *p* < 0.001) and 63.43 ± 6.15% (^&&&^ *p* < 0.001) in the MTT reduction and NR uptake assays, respectively (Figure 3). As 0.5% DMSO served as the vehicle for *rac*-**2b**, comparison with the corresponding vehicle control ensured that the observed biological effects were attributable to the compound rather than to vehicle-associated effects.

Overall, *rac*-**2b** attenuated PQ-induced cytotoxicity, as reflected by improved MTT reduction and NR uptake values, indicating preservation of cellular metabolic activity and lysosomal function.

Similarly, *rac*-**2a** also exhibited a significant neuroprotective effect, although this was only evident in the NR uptake assay (Figure 3B). Compared with PQ-treated cells (51.32 ± 4.52%, ****p < 0.0001), treatment with *rac*-**2a** increased NR uptake to 55.75 ± 4.62% (^#^ *p* < 0.05) and 61.44 ± 7.49% (^##^ *p* < 0.01) at 10 and 100 μM, respectively.

Compounds **1** and *rac*-**1** also produced a partial neuroprotective effect, with statistically significant improvements observed exclusively in the NR uptake assay (Figure 3B). At 10 and 100 μM, compound **1** increased NR uptake to 57.05 ± 5.13% (^##^ *p* < 0.01) and 61.31 ± 6.63% (^####^ *p* < 0.0001), respectively; the corresponding NR uptake values for *rac*-**1** were 58.64 ± 6.32% (^#^ *p* < 0.05) and 68.64 ± 10.18% (^##^ *p* < 0.01). These findings suggest that both compounds preferentially help preserve lysosomal functionality, with limited effects on mitochondrial metabolic activity under PQ-induced stress. Notably, no significant differences were observed between enantiomerically pure rasagiline (**1**) and its racemic mixture (*rac*-**1**), supporting the use of the racemic derivatives *rac*-**2a** and *rac*-**2b** throughout this study.

Overall, *rac*-**2a** and particularly *rac*-**2b** displayed the most consistent neuroprotective profiles against PQ-induced toxicity. Whereas *rac*-**2a** exerted a significant protective effect only in the NR uptake assay, *rac*-**2b** significantly improved cell viability in both the MTT reduction and NR uptake assays, indicating protection of both mitochondrial metabolic activity and lysosomal integrity. Although all tested compounds significantly attenuated PQ-induced lysosomal damage in the NR uptake assay at both 10 and 100 μM, the magnitude and consistency of the response differed among compounds. Collectively, these findings identify *rac*-**2b** as the most promising derivative in this series, exhibiting greater neuroprotective activity than rasagiline under PQ-induced stress.

## MAO Inhibition Assay

To determine whether the observed neuroprotective effects were related to MAO inhibition, enzymatic assays were performed to evaluate their inhibitory activity. In these experiments, **1**, *rac*-**1**, and isosteres *rac*-**2a** and *rac*-**2b** were tested against human (*h*)­MAO-A and (*h*)­MAO-B using a time-dependent Amplex Red oxidation by horseradish peroxidase (AR/HRP) assay. For *h*MAO-B, inhibition was assessed after 5, 15, and 30 min preincubation, whereas for *h*MAO-A, a 15 min preincubation period was used, as previously described.^57,58^

As expected, both **1** (commercially available enantiomerically pure rasagiline) and its racemate, *rac*-**1**, displayed potent and time-dependent inhibition of *h*MAO-B (Table 1 and Figure 4). Compound **1** exhibited submicromolar potency at short preincubation times (IC_50_ = 0.375 μM at 5 min), which further increased upon prolonged preincubation (IC_50_ = 0.098 μM at 30 min), consistent with a time-dependent inhibition mechanism (Table 1 and Figure 4). The racemate *rac*-**1** showed a similar trend, albeit with approximately 2-fold lower potency across all time points (IC_50_ = 0.744 μM at 5 min; 0.184 μM at 30 min), reflecting the contribution of the inactive enantiomer.^23^

**Table 1:** Inhibitory Activity (IC_50_, μM) of Compounds **1**, *rac*-**1**, *rac*-**2a**, and *rac*-**2b** against MAO Isoforms (*h*MAO-A and *h*MAO-B), Determined Using the AR/HRP Assay at Different Preincubation Times

Compound	*h*MAO-A IC_50_ (μM)	*h*MAO-B IC_50_ (μM)
**1**	15 min: 50.35 ± 9.27	5 min: 0.375 ± 0.027
		15 min: 0.186 ± 0.013
		30 min: 0.098 ± 0.006
*rac*-**1**	15 min: 90.05 ± 19.29	5 min: 0.744 ± 0.063
		15 min: 0.373 ± 0.020
		30 min: 0.184 ± 0.011
*rac*-**2a**	15 min: 37.48 ± 13.81	5 min: 57.73 ± 10.94
		15 min: 71.54 ± 16.09
		30 min: 66.56 ± 12.63
*rac*-**2b**	n.a.^a^	5 min: 74.74 ± 15.19
		15 min: 49.27 ± 6.18
		30 min: 47.45 ± 13.96

a n.a., not active (residual activity: RA at 100 μM > 50%)

**Figure 4:**
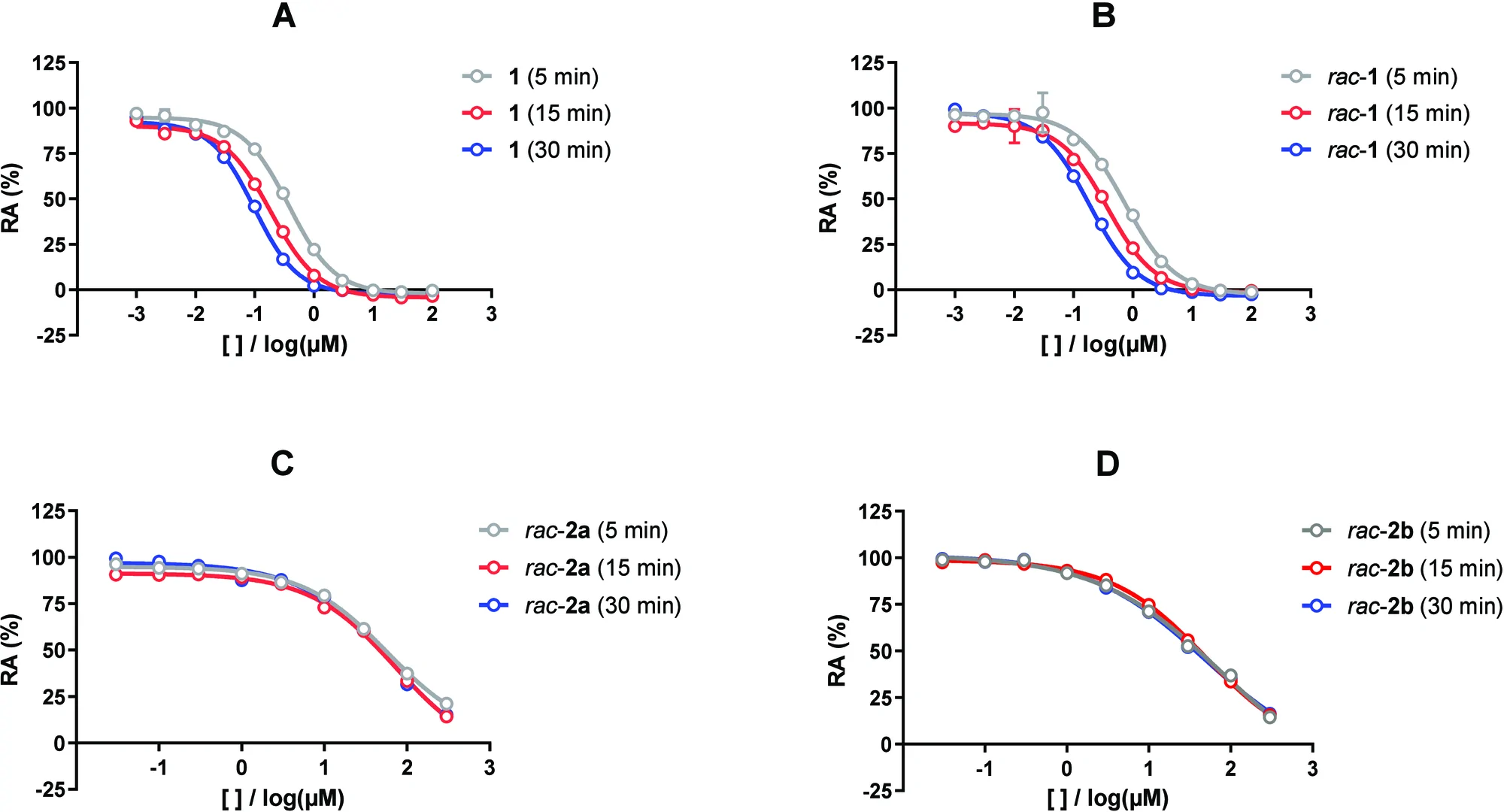
Concentration–response curves for inhibition of *h*MAO-B by compounds **1 (A)**, *rac*-**1 (B)**, *rac*-**2a (C)**, and *rac*-**2b (D)**, determined using the AR/HRP assay at different preincubation times (5, 15, and 30 min).

In contrast, both reference compounds displayed weak inhibition of *h*MAO-A, with IC_50_ values in the tens of micromolar range (50.35 μM for **1** and 90.05 μM for *rac*-**1**, Table 1), confirming their marked selectivity toward *h*MAO-B.

A markedly different profile was observed for the novel analogs *rac*-**2a** and *rac*-**2b**. For *h*MAO-B, both compounds exhibited only weak inhibition, with IC_50_ values in the high micromolar range (approximately 47–75 μM) and, importantly, no significant time-dependent increase in potency (Table 1, Figure 4). When compared to the racemic reference *rac*-**1**, this corresponds to a loss of activity of approximately 2 orders of magnitude, shifting from submicromolar to tens of micromolar IC_50_ values. Relative to **1**, the decrease reaches nearly 3 orders of magnitude at longer preincubation times.

A similar trend was observed for *h*MAO-A (Table 1). While *rac*-**1** already displays weak inhibition (IC_50_ = 90.05 μM), *rac*-**2a** shows only marginally improved activity (IC_50_ = 37.48 μM), remaining within the same order of magnitude, and *rac*-**2b** exhibited no detectable inhibition under the experimental conditions. Overall, no meaningful enhancement of *h*MAO-A inhibition is observed for the new analogs, and all compounds remain weak inhibitors of this isoform.

Importantly, comparison of *rac*-**2a** and *rac*-**2b** with the racemic reference *rac*-**1** indicates that the pronounced loss of *h*MAO-B inhibitory activity arises from the structural modifications introduced, rather than stereochemical effects (Table 1, Figure 4). At the same time, *h*MAO-A inhibition remains weak across the series (Table 1). Furthermore, the absence of time-dependent inhibition for both compounds, together with their markedly reduced *h*MAO-B inhibitory potency, indicates that they do not display the characteristic behavior of mechanism-based MAO-B inhibition observed for rasagiline under the experimental conditions employed.^33^

The substantially attenuated inhibitory activity observed for the azaindane derivatives may be rationalized based on structural features of MAO-B. Crystallographic analysis (PDB: 1S2Q) reveals a predominantly hydrophobic active site composed of a substrate cavity and an entrance cavity, connected by the gating residue Ile199.^17^ Compared to rasagiline, the introduction of a nitrogen atom into the aromatic ring increases polarity and may perturb favorable interactions within the predominantly hydrophobic MAO-B active site.^17^ Given the compact nature of the binding pocket and its sensitivity to subtle structural changes, this modification may affect ligand recognition and/or subsequent steps required for irreversible inhibition, including enzyme-catalyzed oxidation of the propargylamine moiety followed by covalent modification of the FAD cofactor. Thus, while these considerations provide a plausible structural rationale for the experimentally observed reduction in MAO-B inhibition, the present data do not establish which step of this multistep process is primarily affected by the ring nitrogen.^17,33^

Collectively, these results demonstrate that *rac*-**2a** and *rac*-**2b** lack the potent, time-dependent *h*MAO-B inhibition characteristic of rasagiline, while maintaining only weak or negligible activity toward *h*MAO-A across the series. This corresponds to a reduction of approximately 2 to 3 orders of magnitude in *h*MAO-B inhibitory potency relative to rasagiline. Overall, these findings indicate that the indane-to-azaindane isosteric modification effectively abolishes potent *h*MAO-B inhibition, suggesting that *rac*-**2a** and *rac*-**2b** are unlikely to exert their biological effects through MAO inhibition.

Overall, a straightforward and reliable synthetic route for the assembly of rasagiline isostere *rac*-**2a** was established based on the direct, regioselective oxidation of CPP to afford ketone **6a** as a key intermediate, using ^*t*^BuOOH (in nonane) catalyzed by Mn­(OAc)_2_. Despite the moderate chemical yield obtained for ketone **6a** under this protocol (42%), this value is within the range reported for photochemical and electrochemical processes (41–57%). Importantly, the transformation is operationally simple, fully regioselective, facilitates chromatographic purification, and does not require any specialized setup or equipment.

From a medicinal chemistry perspective, this work expands previous studies on rasagiline (**1**) in PQ-induced neurotoxicity by showing preservation of lysosomal integrity, as assessed by the NR uptake assay, in dopaminergic-differentiated SH-SY5Y cells. Within this context, *rac*-**2a** displayed a neuroprotective profile comparable to rasagiline, whereas its regioisomer *rac*-**2b** exhibited superior neuroprotective activity in the PQ-insulted differentiated SH-SY5Y cellular model employed, indicating that the indane-to-azaindane strategy is highly dependent on the position of nitrogen substitution. In contrast, both regioisomers markedly reduced MAO-B inhibitory activity, regardless of substitution pattern. Collectively, these results highlight a clear divergence between neuroprotective efficacy and MAO inhibition governed by azaindane regiochemistry. Notably, the data support the conclusion that the neuroprotective effects observed for *rac*-**2a** and *rac*-**2b** are independent of MAO-B inhibition, highlighting a pharmacological dissociation between these activities and suggesting a potentially safer neuroprotective mechanism, free from MAO-related adverse effects associated with tyramine-mediated interactions.

Altogether, these findings support the progression of this project toward the enantioselective preparation of these rasagiline isosteres, paving the way for the development of new neuroprotective leads to halt the progression of neurodegenerative disorders such as PD.

## Supplementary Material



## Notes

*In silico* prediction of blood-brain barrier permeability, detailed synthetic procedures and copies of ^1^H, ^13^C­{^1^H}, DEPT-135 NMR spectra for compounds **4**, **5**, **6a**, **6b**, *rac*-**1**, *rac*-**2a**, and *rac*-**2b**, and HRMS data for *rac*-**2a** (PDF)

## #

Biomedicine Department, Faculty of Medicine, University of Porto, 4200–319 Porto, Portugal
