# Supplementary material for: Decoupling Neuroprotection from MAO Inhibition via Indane-to-Azaindane Bioisosteric Replacement of Rasagiline
Source: ACS Med Chem Lett. 2026 Sep 12;17(10):2251–9. doi: 10.1021/acsmedchemlett.6c00407 (PMC13656277; doi:10.1021/acsmedchemlett.6c00407)
Supplement: Supplementary file 1 [file ml6c00407_si_001.pdf]

Supporting Information for:

Decoupling Neuroprotection from MAO Inhibition via Indane-to-Azaindane Bioisosteric Replacement of Rasagiline

Ivo E. Sampaio-Dias,[a,*] Sara C. Silva-Reis,[a,b] Xavier C. Correia,[a] Hugo F. Costa-Almeida,[a] Maria B. Igreja-Cardoso,[a] Gabriel M. Azevedo,[a] Vera M. Costa,[b,c,#] Damijan Knez,[d] Stanislav Gobec,[d] and José E. Rodríguez-Borges[a]

[a]LAQV/REQUIMTE, Department of Chemistry and Biochemistry, Faculty of Sciences, University of Porto, R. Campo Alegre s/n, 4169-007 Porto, Portugal.

[b]UCIBIO/REQUIMTE, Laboratory of Toxicology, Faculty of Pharmacy, University of Porto, 4050-313 Porto, Portugal.

[c]Associate Laboratory i4HB - Institute for Health and Bioeconomy, Faculty of Pharmacy, University of Porto, 4050-313 Porto, Portugal.

[d]University of Ljubljana, Faculty of Pharmacy, Aškerčeva 7, 1000 Ljubljana, Slovenia.

[#]Present address: Biomedicine Department, Faculty of Medicine, University of Porto, 4200-319 Porto, Portugal.

***Corresponding author e-mail**:
ivdias@fc.up.pt (Ivo E. Sampaio-Dias)

Table of Contents

1. *In Silico* Prediction of Blood-Brain Barrier Permeability S2
2. Organic Chemistry: Experimental Section S3
   - 2.1. General aspects S3
   - 2.2. Apparatus S3
   - 2.3. Reaction optimization for the oxidation of CPP to ketone **6a** S4
   - 2.4. Experimental Protocols S5
3. NMR Spectra S8
4. HRMS Spectrum of *rac*-**2a** S15
5. Toxicological Assays: Experimental Section S15
6. *h*MAO Inhibition Assays S17
7. References S18

1. *In Silico* Prediction of Blood-Brain Barrier Permeability

Selected physicochemical and pharmacokinetic properties of rasagiline (**1**) and azaindane derivatives *rac*-**2a** and *rac*-**2b** were evaluated *in silico* using SwissADME (Table S1). Blood-brain barrier permeation was assessed using the BOILED-Egg model, together with the predicted propensity of the compounds to act as P-glycoprotein (P-gp) substrates (Figure S1).

Table S1. Predicted physicochemical and CNS-related properties of rasagiline (**1**) and azaindane derivatives *rac*-**2a** and *rac*-**2b** using SwissADME.[a]

Compound	MW (g/mol)	TPSA (Å^2)	Consensus Log *P*	BBB permeant	P-gp substrate
**1**	171.24	12.03	2.23	Yes	No
rac-**2a**	172.23	24.92	1.38	Yes	No
rac-**2b**	172.23	24.92	1.38	Yes	No

[a]MW, molecular weight; TPSA, topological polar surface area; BBB, blood-brain barrier; P-gp, P-glycoprotein. Physicochemical and pharmacokinetic properties were predicted using the SwissADME web tool (www.swissadme.ch).

The introduction of the ring nitrogen increases the polar character of the azaindane derivatives relative to rasagiline, as reflected by the increase in TPSA and decrease in consensus Log P. Nevertheless, all three compounds are predicted to passively permeate the BBB (BBB+) and not to be P-gp substrates (PGP−), as shown in Figure S1. Thus, despite the increased polarity associated with the indane-to-azaindane replacement, the predicted physicochemical profiles of *rac*-**2a** and *rac*-**2b** remain compatible with CNS accessibility.

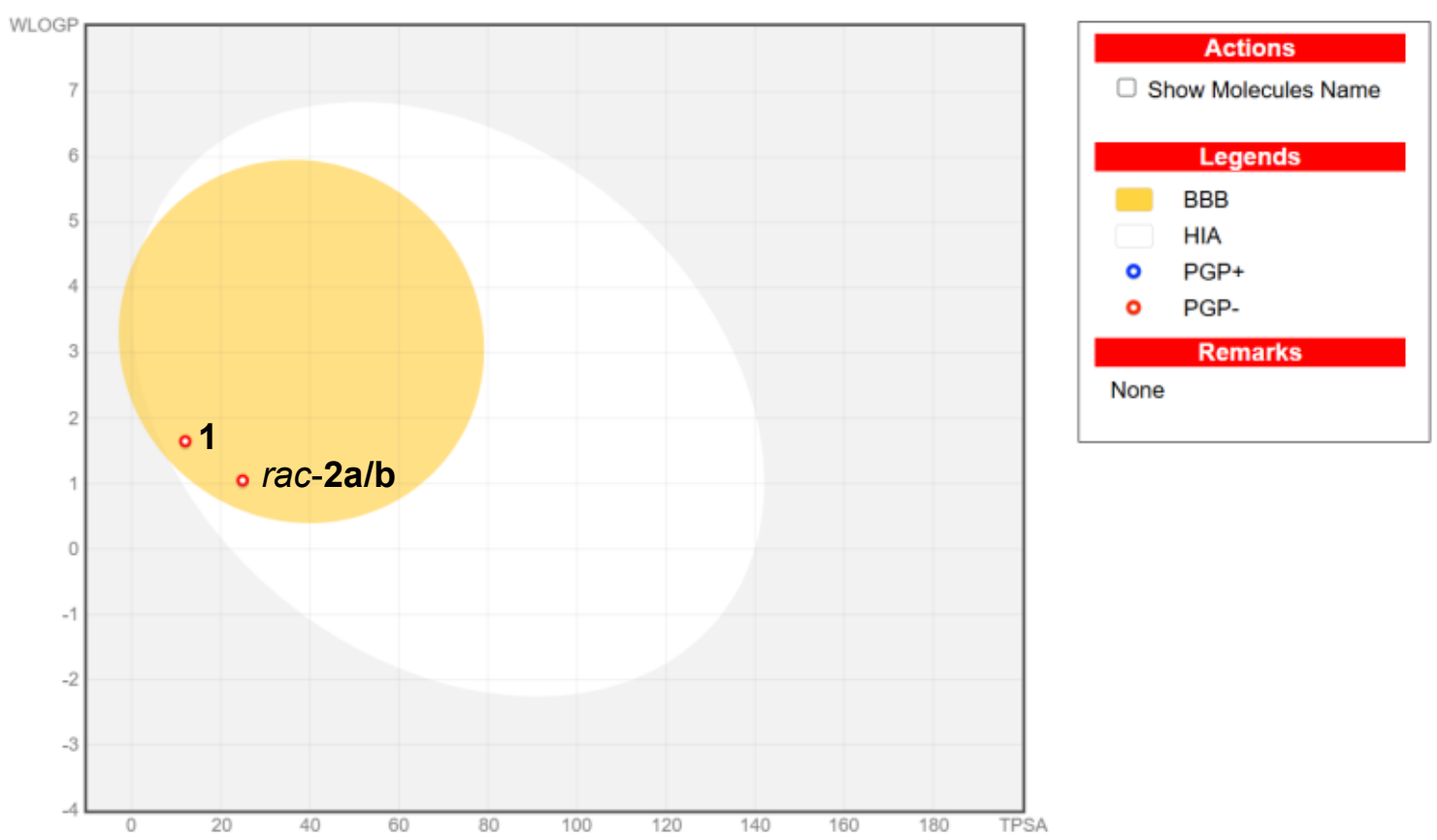

Figure S1. BOILED-Egg representation of rasagiline (**1**) and azaindane derivatives *rac*-**2a** and *rac*-**2b** generated using SwissADME. Compounds located within the yellow region (yolk) are predicted to passively permeate the blood-brain barrier (BBB), whereas red circles indicate compounds predicted not to be substrates of P-glycoprotein (PGP−). The data points corresponding to *rac*-**2a** and *rac*-**2b** overlap due to their identical TPSA and WLOGP values.

2. Organic Chemistry: Experimental Section

2.1. General Aspects

All reagents and solvents were of commercial reagent grade and used without further purification: 6,7-dihydro-5*H*-cyclopenta[*b*]pyridine was purchased from Fluorochem (Derbyshire, UK); Pd/C (10 wt. % loading), $Mn(OTf)_2$, $Mn(OAc)_2$, (carbethoxymethylene)triphenylphosphorane, propargylamine, ^{t}BuOOH (70% in H_2O), ^{t}BuOOH (5.5 M in nonane), and $NaBH(OAc)_3$, were obtained from Sigma-Aldrich (Algés, Portugal). High-purity grade silica gel used for flash chromatography (Merck Silica gel 60, 230-400 mesh ASTM) and pre-coated aluminum silica gel plates used for analytical TLC (Merck Silica gel 60 F_{254}) were obtained from Merck (Darmstadt, Germany). TLC plates were analyzed under UV radiation (254 and 366 nm) and/or by using a solution of phosphomolybdic acid in ethanol, requiring gentle heating for visualization.

2.2. Apparatus

HRMS analyses were performed at CEMUP (Centro de Materiais da Universidade do Porto) using an LTQ Orbitrap XL™ hybrid mass spectrometer (Thermo Fisher Scientific, Bremen, Germany) equipped with an electrospray ionization (ESI) source and controlled by Xcalibur 2.1.0 and LTQ Tune Plus 2.5.5 software. The capillary voltage and the temperature were set to 3.1 kV and 275 °C, respectively, and the sheath gas was set at 6 (arbitrary units) according to the software settings. The capillary and the tube lens voltage were set to 35 V and 110 V, respectively. NMR spectra were recorded at CEMUP with a Bruker Avance III 400 at 400.15 MHz for ^{1}H and 100.62 MHz for ^{13}C. Chemical shifts (δ) are reported in ppm and referenced to the residual protic solvent signals ($CDCl_3$: δ_H = 7.26) in the ^{1}H NMR spectra and the deuterated solvent signals ($CDCl_3$: δ_C = 77.16) in the $^{13}C\{^{1}H\}$ NMR spectra. Coupling constants (*J*) were reported in Hz. Melting points were determined using a STUART Scientific SMP1 apparatus and are uncorrected. Solvents were evaporated under reduced pressure using a Büchi rotavapor RE-111.

2.3. Reaction optimization for the oxidation of CPP to ketone 6a:

Table S2. Optimization of the $Mn(OAc)_2$-catalyzed oxidation of CPP to ketone **6a** using ^{t}BuOOH in nonane.

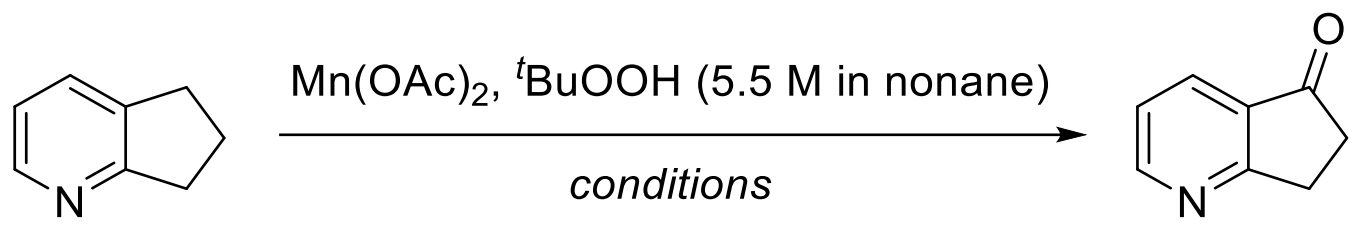

CPP **6a**

Entry	$Mn(OAc)_2$ (mol%)	^{t}BuOOH (equiv.)	Time / h	Temperature / ºC	Yield[a] / %
1	none	5	24	20	n.d.
2	0.5	5	24	20	12
3	0.5	10	24	20	10
4	0.5	5	48	20	18
5	0.5	5	24	50	19
6	5	5	24	20	32
7	5	5	48	20	42
8	5	5	48	50	40
9	5	5	72	20	32
10	10	5	24	20	39

[a]Isolated yield; n.d. – not detected.

2.4. Experimental Protocols

2.4.1. Synthesis of *rac*-2b. This compound was synthesized from CPP according to the previously reported sequence, which involves intermediates **8**-**10** and **6b**. Analytical and spectroscopic data of *rac*-**2b** are in full agreement with literature reports.[2,3]

2.4.2. Synthesis of ethyl (*E*)-3-(pyridin-2-yl)acrylate (4).

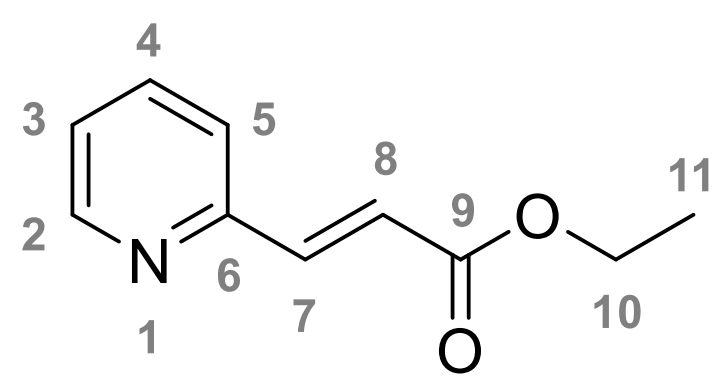

To a solution of pyridine-2-carbaldehyde (1.0008 g, 9.3437 mmol) in CH_2Cl_2 was added (carbethoxymethylene)triphenylphosphorane (3.9061 g, 11.212 mmol), and the reaction mixture was kept under magnetic stirring for 24 h. The volatiles were then removed under reduced pressure, and the crude residue was purified by flash column chromatography using EtOAc as eluent to afford acrylate **4** (1.3577 g, 82%) as a clear oil. **R*f***: 0.82 (EtOAc). **^{1}H NMR (400 MHz, $CDCl_3$) *δ* ppm:** 8.58 (ddd, *J* = 4.8, 2.0, 1.0 Hz, 1H, H-2), 7.68 – 7.59 [7.63 (d, *J* = 15.8 Hz), 2H, H-4 + H-7], 7.37 (dt, *J* = 7.8, 1.1 Hz, 1H, H-5), 7.20 (ddd, *J* = 7.6, 4.8, 1.2 Hz, 1H, H-3), 6.86 (d, *J* = 15.7 Hz, 1H, H-8), 4.22 (q, *J* = 7.2 Hz, 2H, H-10), 1.28 (t, *J* = 7.2 Hz, 3H, H-11). **^{13}C{^{1}H} and DEPT-135 NMR (101 MHz, $CDCl_3$) *δ* ppm:** 166.7 (C, C-9), 153.0 (C, C-6), 150.1 (CH, C-2), [143.3 (CH), 136.7 (CH), 124.2 (CH), 124.1 (CH), 122.5 (CH), C-3 + C-4 + C-5 + C-7 + C-8], 60.6 (CH_2, C-10), 14.3 (CH_3, C-11).

2.4.3. Synthesis of ethyl 3-(pyridin-2-yl)propanoate (5).

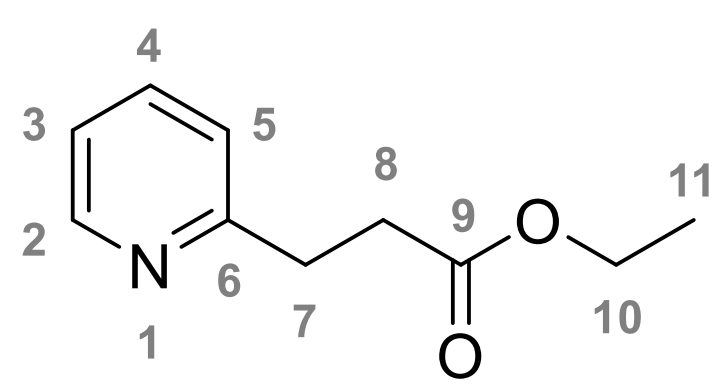

To a solution of **4** (1.3020 g, 7.3476 mmol) in EtOAc (20 mL), catalytic amounts of Pd/C (10% w/w) were added, and the reaction vessel was purged with H_2 and maintained under an H_2 atmosphere (balloon). The resulting black suspension was left under magnetic stirring at 25 ºC until complete consumption of the starting material (*ca*. 1 h, TLC). After that, the suspension was filtered through a Celite® pad and washed with EtOAc (3 x 20 mL). The combined filtrates were concentrated under reduced pressure, and the oily residue was purified by flash column chromatography using hexane/EtOAc (1:2) as eluent to afford saturated ester **5** (1.3037 g, 99%) as a clear oil. **R*f***: 0.61 (Hexane/EtOAc 1:2). **^{1}H NMR (400 MHz, $CDCl_3$) *δ* ppm:** 8.48 (ddd, *J* = 4.9, 1.9, 0.9 Hz, 1H, H-2), [7.54 (dt, *J* = 7.6, 1.8 Hz, 1H), 7.14 (d, *J* = 7.8 Hz, 1H), H-3 + H-4], 7.07 (ddd, *J* = 7.6, 4.9, 1.3 Hz, 1H, H-5), 4.08 (q, *J* = 7.2 Hz, 2H, H-10), [3.07 (t, *J* = 7.5 Hz, 2H), 2.76 (t, *J* = 7.5 Hz, 2H), H-7 + H-8], 1.18 (t, *J* = 7.2 Hz, 3H, H-11). **^{13}C{^{1}H} and DEPT-135 NMR (101 MHz, $CDCl_3$) *δ* ppm:** 173.1 (C, C-9), 160.2 (C, C-6), 149.4 (CH, C-2), [136.4 (CH), 123.0 (CH), 121.4 (CH), C-3 + C-4 + C-5], 60.4 (CH_2, C-10), [33.5 (CH_2), 33.0 (CH_2), C-7 + C-8], 14.2 (CH_3, C-11).

2.4.4. Synthesis of 6,7-dihydro-5*H*-cyclopenta[*b*]pyridin-5-one (6a).

Protocol A (*adapted*)[1]: To a round-bottom flask were consecutively added CPP (2.9913 g, 25.102 mmol), $Mn(OTf)_2$ (0.0445 g, 0.126 mmol), ^tBuOOH (70% in H_2O, 17.3 mL, 126 mmol), and H_2O (125 mL). The reaction mixture was left under magnetic stirring at 25 ºC for 72 h. After that, sodium thiosulfate pentahydrate was added until the KI-starch test paper did not change its color. The mixture was then transferred into a separatory funnel and extracted with EtOAc (3 x 100 mL). The combined organic extracts were dried over anhydrous Na_2SO_4, filtered, and concentrated under reduced pressure. The oily residue obtained was purified by flash column chromatography using Et_2O as eluent to afford ketone **6a** (0.5348 g, 16%) along with ketone **6b** (0.0668 g, 2%), both as beige solids.

Protocol B (*this work*): To a solution of CPP (1.0082 g, 8.4604 mmol) in dry hexane (10 mL) was added $Mn(OAc)_2$ (0.0732 g, 0.423 mmol), and the reaction mixture was cooled to –20 ºC. ^tBuOOH (5.5 M in nonane, 7.69 mL, 42.3 mmol) was then slowly added dropwise over 20 min, and the resulting mixture was left under magnetic stirring at 20 ºC for 48 h. After that, a saturated aqueous solution of $NaHCO_3$ (20 mL) was added, followed by sodium thiosulfate pentahydrate (*ca*. 1.5 g). The mixture was transferred into a separatory funnel and extracted with EtOAc (3 x 50 mL). The combined organic extracts were dried over anhydrous Na_2SO_4, filtered, and concentrated under reduced pressure. The oily residue obtained was purified by flash column chromatography using Et_2O as eluent to afford ketone **6a** (0.4731 g, 42%) as a beige solid.

Spectroscopic data for **6a**:

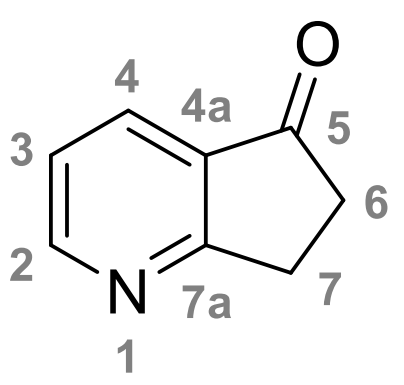

mp: 62-63 ºC. **R*f***: 0.30 (Et_2O). **^{1}H NMR (400 MHz, $CDCl_3$) *δ* ppm:** 8.80 (dd, *J* = 4.8, 1.7 Hz, 1H, H-2), 8.02 (dd, *J* = 7.7, 1.7 Hz, 1H, H-4), 7.32 (dd, *J* = 7.7, 4.8 Hz, 1H, H-3), 3.35 – 3.21 (m, 2H, H-7), 2.84 – 2.71 (m, 2H, H-6). **^{13}C{^{1}H} and DEPT-135 NMR (101 MHz, $CDCl_3$) *δ* ppm:** 204.9 (C, C-5), 174.4 (C, C-7a), 155.7 (CH, C-2), 131.9 (CH, C-4), 130.3 (C, C-4a), 122.5 (CH, C-3), 35.8 (CH_2, C-6), 28.7 (CH_2, C-7).

Spectroscopic data for **6b**:

mp: 98-101 ºC. **R*f***: 0.30 (EtOAc). **^{1}H NMR (400 MHz, $CDCl_3$) *δ* ppm:** 8.80 – 8.75 (m, 1H, H-2), 7.89 (ddt, *J* = 7.8, 1.5, 0.8 Hz, 1H, H-4), 7.45 (dd, *J* = 7.9, 4.6 Hz, 1H, H-3), 3.21 – 3.14 (m, 2H, H-6), 2.80 – 2.73 (m, 2H, H-5). **^{13}C{^{1}H} and DEPT-135 NMR (101 MHz, $CDCl_3$) *δ* ppm:** 205.3 (C, C-7), 154.1 (C, C-7a), 150.8 (CH, C-2), 149.5 (C, C-4a), 135.3 (CH, C-4), 127.3 (C, C-3), 34.9 (CH_2, C-6), 24.4 (CH_2, C-5).

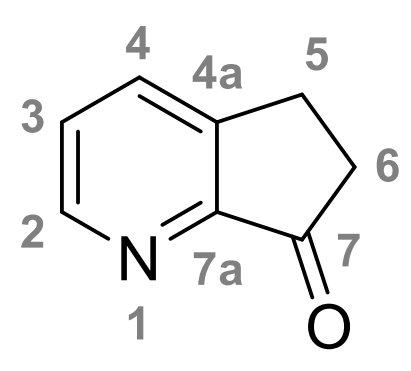

2.4.5. Synthesis of (*R/S*)-*N*-(prop-2-yn-1-yl)-6,7-dihydro-5*H*-cyclopenta[*b*]pyridin-5-ylamine (*rac*-2a).

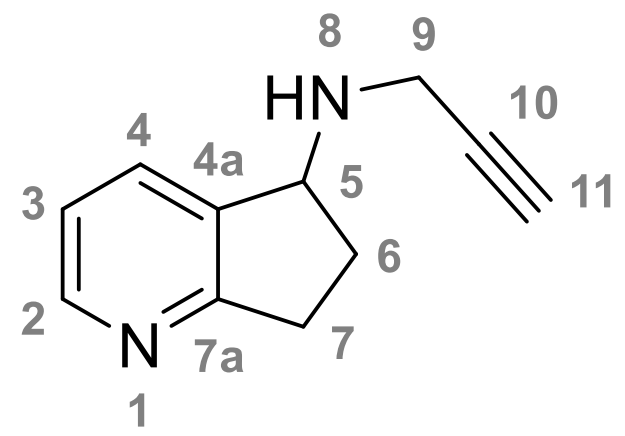

To a solution of ketone **6a** (0.1727 g, 1.297 mmol) in CH_2Cl_2 (20 mL), previously purged with Ar, was added propargylamine (0.16 mL, 2.5 mmol), and the reaction mixture was left under magnetic stirring for 24 h. After that, $NaBH(OAc)_3$ (0.5400 g, 2.548 mmol) was added, and the reaction mixture was stirred for a further 24 h. The mixture was filtered, and the filtrate was concentrated under reduced pressure. The oily residue obtained was purified by flash column chromatography using DCM/MeOH 10:1 as eluent to afford *rac*-**2a** (0.1698 g, 76%) as a brown oil. **R*f***: 0.28 (DCM/MeOH 10:1).

^{1}H NMR (400 MHz, $CDCl_3$) *δ* ppm: 8.35 (dd, *J* = 5.0, 1.0 Hz, 1H, H-2), 7.60 (d, *J* = 7.5 Hz, 1H, H-4), 7.04 (dd, *J* = 7.6, 5.0 Hz, 1H, H-3), 4.48 – 4.31 (m, 1H, H-5), 3.46 (d, *J* = 2.4 Hz, 2H, H-9), 3.19 – 2.99 (m, 3H, H-6 + NH), 2.94 – 2.81 (m, 1H, H-6), 2.42 (dddd, *J* = 12.8, 8.7, 7.1, 5.5 Hz, 1H, H-7), 2.24 (t, *J* = 2.5 Hz, 1H, H-11), 1.84 (dddd, *J* = 13.0, 8.8, 6.6, 5.5 Hz, 1H, H-7). **^{13}C{^{1}H} and DEPT-135 NMR (101 MHz, $CDCl_3$) *δ* ppm:** 164.8 (C, C-7a), 148.9 (CH, C-2), 137.54 (C, C-4a), 137.5 (CH, C-4), 121.3 (CH, C-3), 81.9 (C, C-10), 71.9 (CH, C-11), 59.9 (CH, C-5), 35.9 (CH_2, C-9), 32.1 (CH_2, C-6), 31.5 (CH_2, C-7). **HRMS (ESI-TOF) *m/z***: $[M+H]^+$ calculated for $[C_{11}H_{13}N_2]^+$: 173.10732; obtained: 173.10814.

2.4.6. Synthesis of *rac*-rasagiline (*rac*-1).

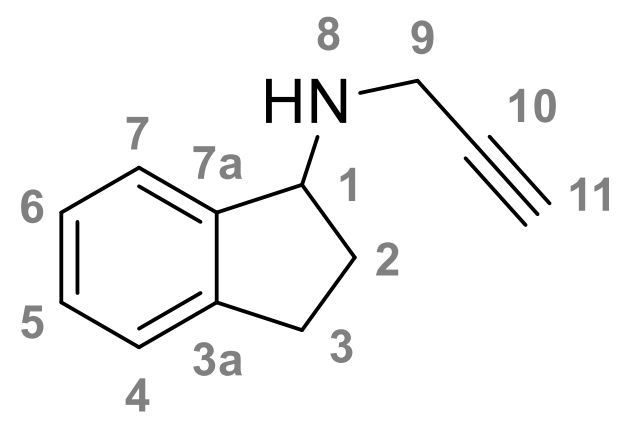

To a solution of **11** (0.2505 g, 1.895 mmol) in CH_2Cl_2 (20 mL), previously purged with Ar, was added propargylamine (0.24 mL, 3.7 mmol), and the reaction mixture was left under magnetic stirring for 24 h. After that, $NaBH(OAc)_3$ (0.8041 g, 3.7940 mmol) was added, and the reaction mixture was stirred for a further 24 h. The mixture was filtered, and the filtrate was concentrated under reduced pressure. The oily residue obtained was purified by flash column chromatography using EtOAc as eluent to afford *rac*-**1** (0.2564 g, 79%) as a pale-yellow oil. **R*f***: 0.31 (Hex/EtOAc 1:1).

^{1}H NMR (400 MHz, $CDCl_3$) *δ* ppm: 7.68 – 6.71 (m, 4H, H-4 + H-5 + H-6 + H-7), 4.50 – 4.17 (m, 1H, H-1), 3.44 (t, *J* = 2.6 Hz, 2H, H-9), 2.96 (ddd, *J* = 14.4, 8.1, 5.9 Hz, 1H, H-2a), 2.82 – 2.66 (m, 1H, H-2b), 2.32 (dddd, *J* = 12.7, 8.3, 6.9, 5.8 Hz, 1H, H-3a), 2.18 (t, *J* = 2.4 Hz, 1H, H-11), 1.89 – 1.67 (m, 1H, H-3b), 1.49 (br s, 1H, NH). **^{13}C{^{1}H} and DEPT-135 NMR (101 MHz, $CDCl_3$) *δ* ppm:** [144.6 (C), 143.9 (C), C-3a + C-7a], [127.8 (CH), 126.4 (CH), 125.0 (CH), 124.3 (CH), C-4 + C-5 + C-6 + C-7], 82.6 (C, C-10), 71.5 (CH, C-11), 62.0 (CH, C-1), 36.3 (CH_2, C-9), [33.4 (CH_2), 30.6 (CH_2), C-2 + C-3].

3. NMR Spectra

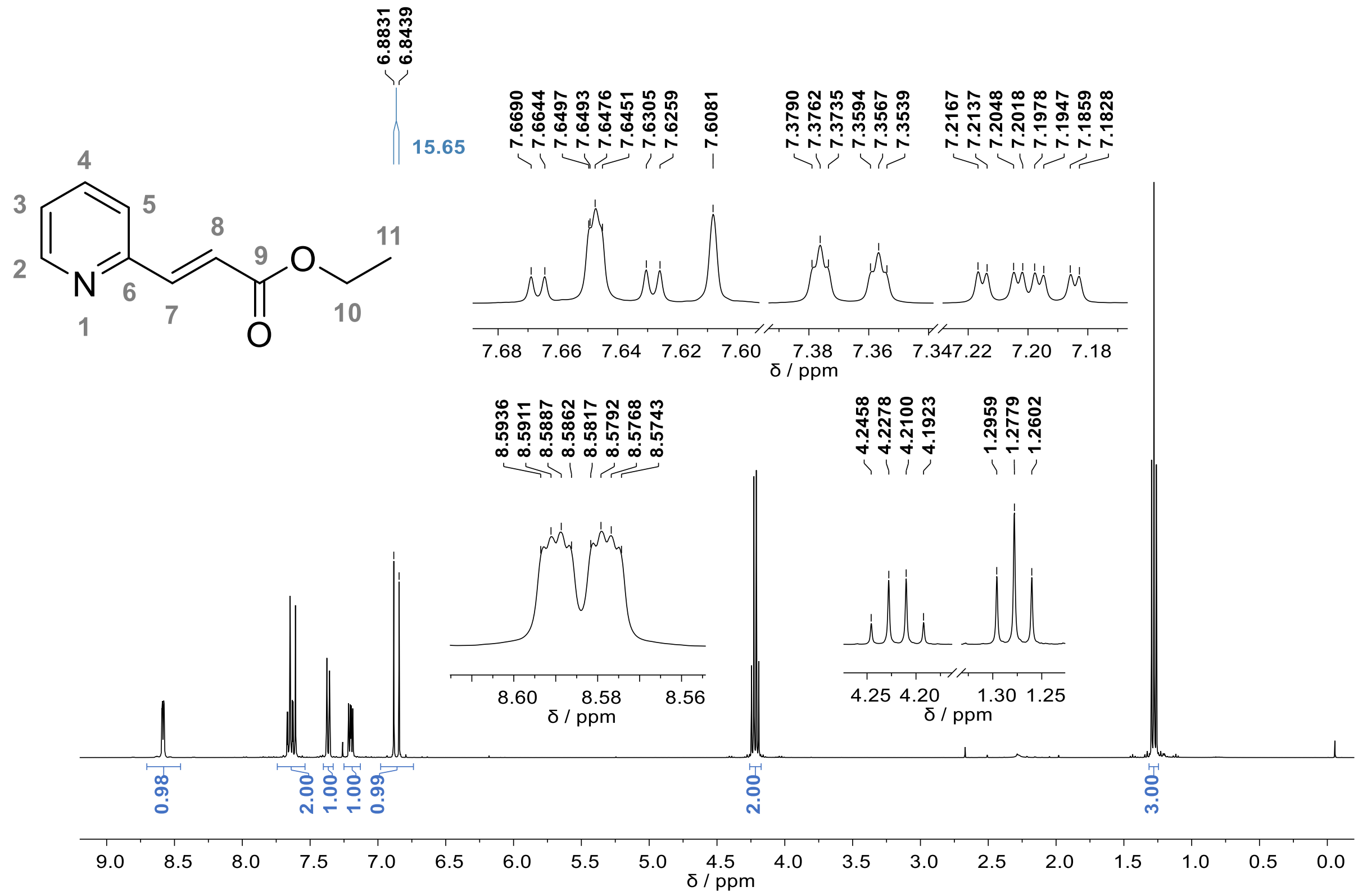

Figure S2. ^{1}H NMR spectrum ($CDCl_3$, 400 MHz) of unsaturated ester **4**.

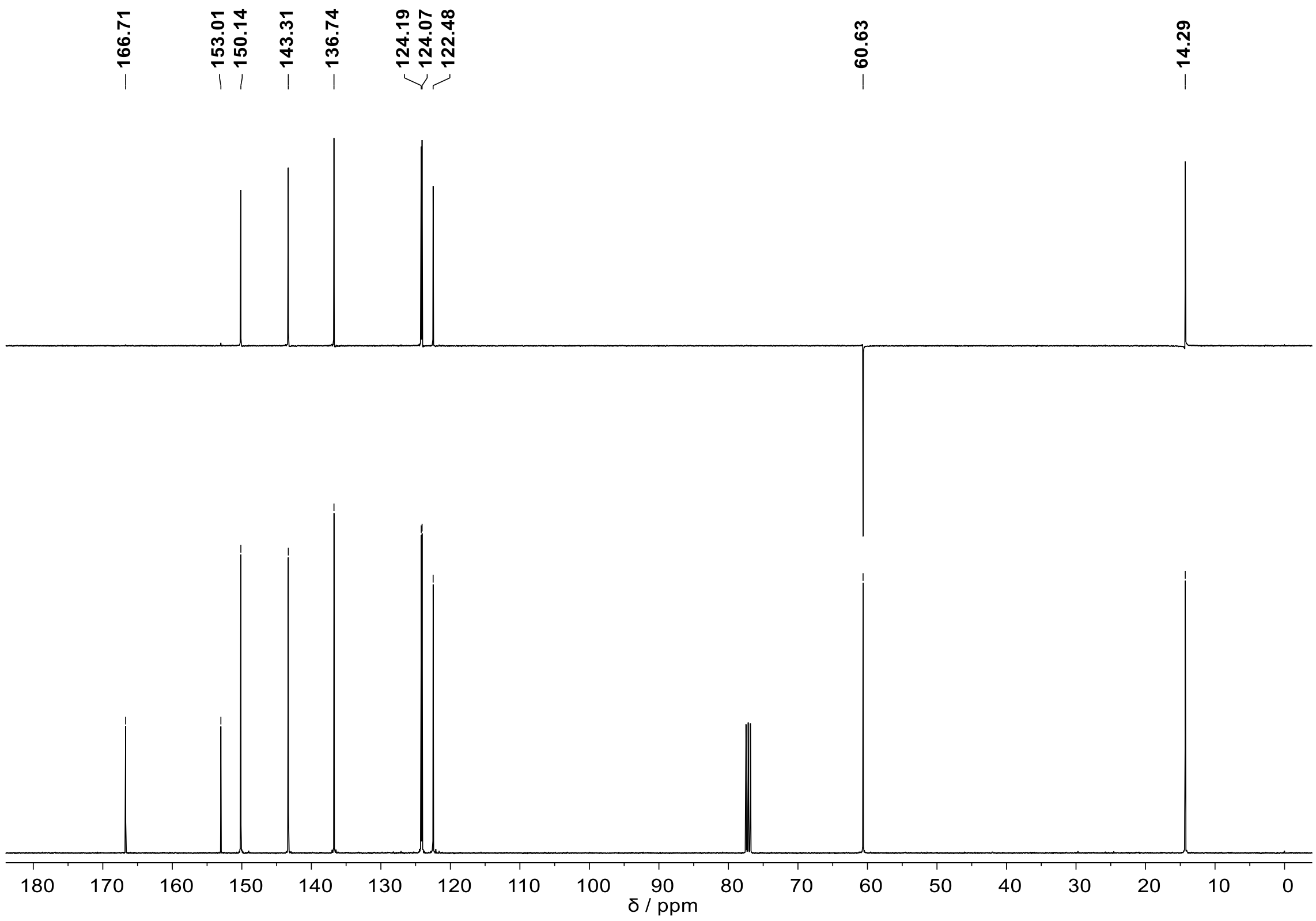

Figure S3. $^{13}C\{^{1}H\}$ and DEPT-135 NMR spectra ($CDCl_3$, 101 MHz) of unsaturated ester **4**.

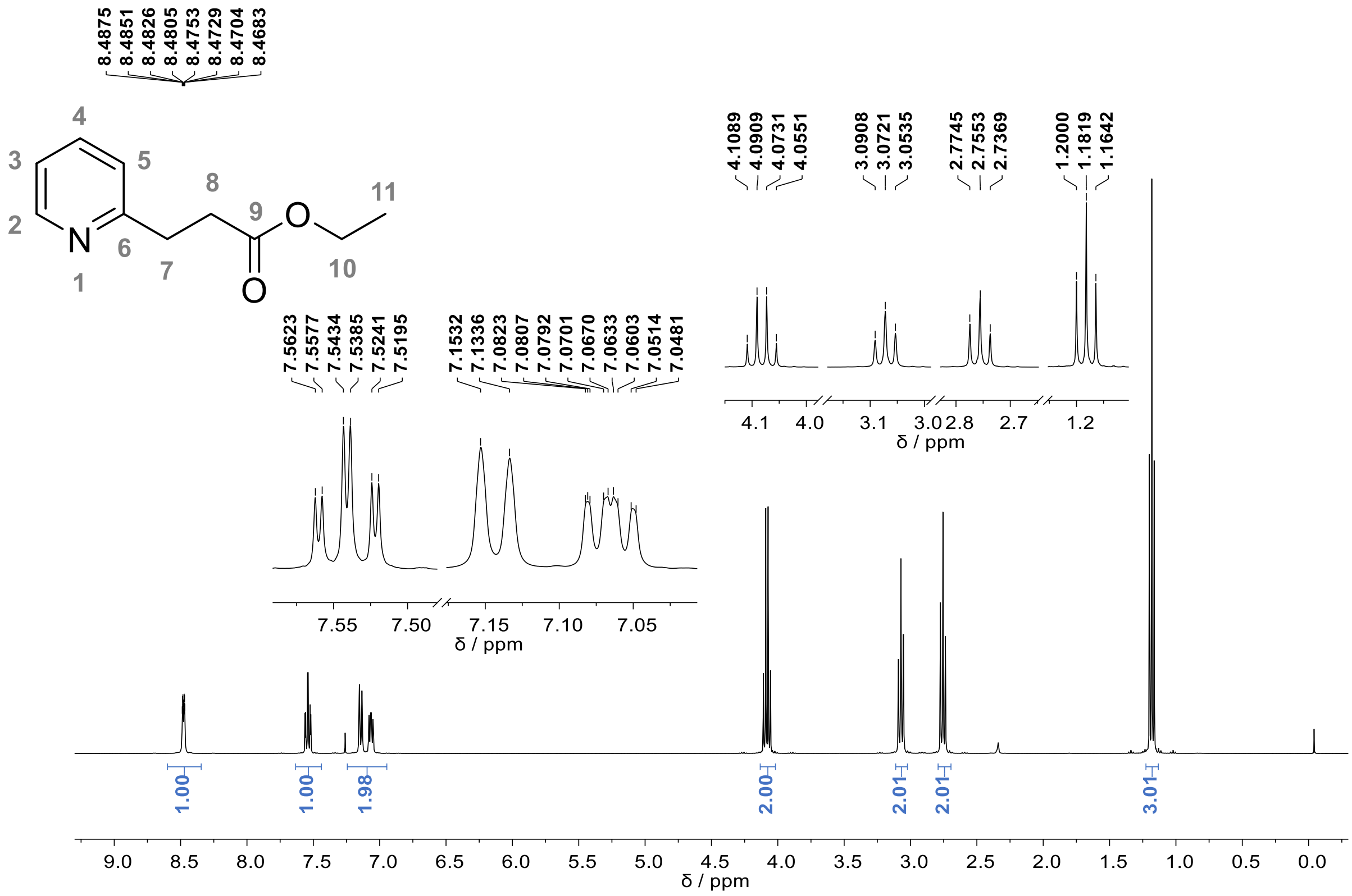

Figure S4. ^{1}H NMR spectrum ($CDCl_3$, 400 MHz) of saturated ester **5**.

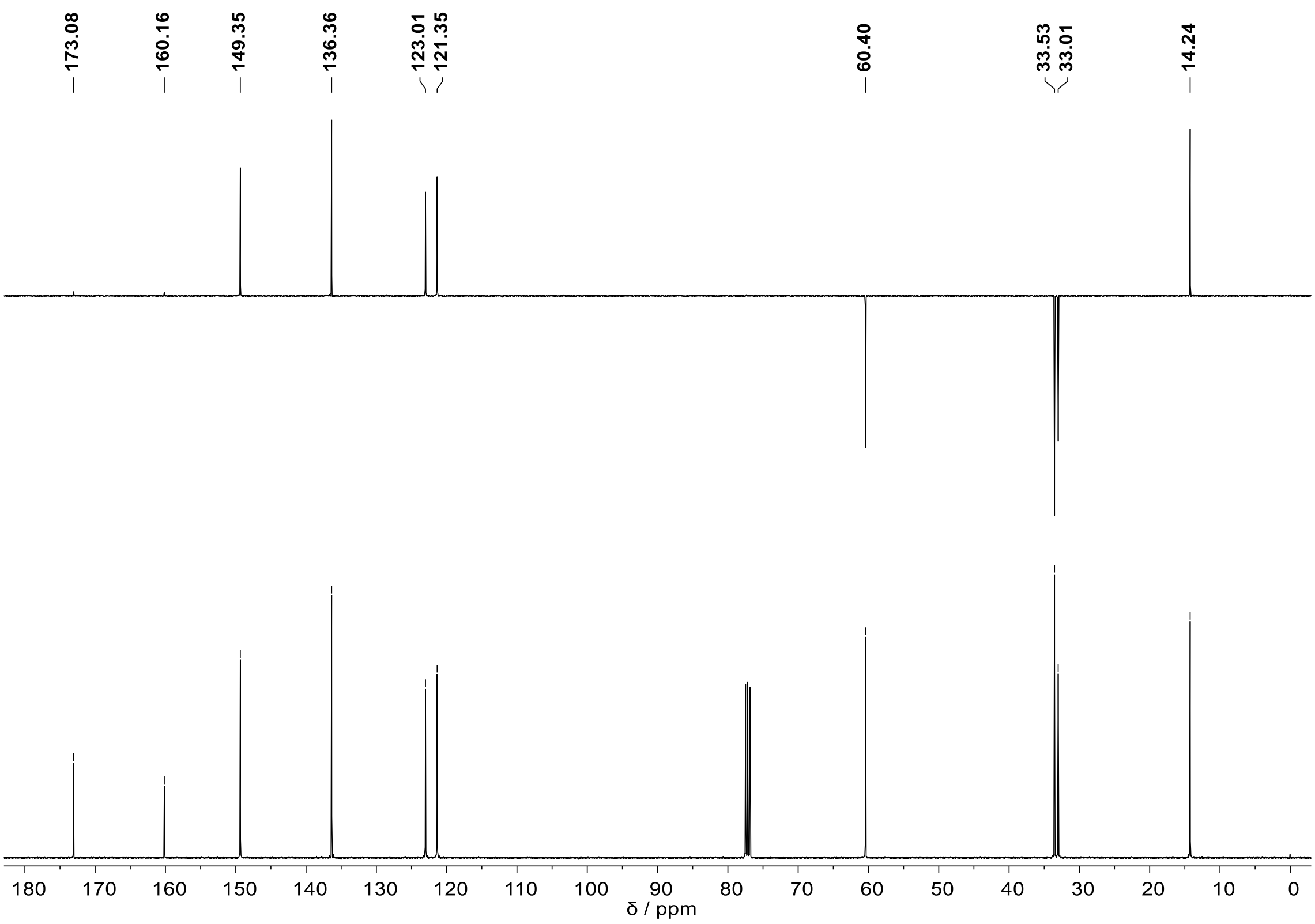

Figure S5. $^{13}C\{^{1}H\}$ and DEPT-135 NMR spectra ($CDCl_3$, 101 MHz) of saturated ester **5**.

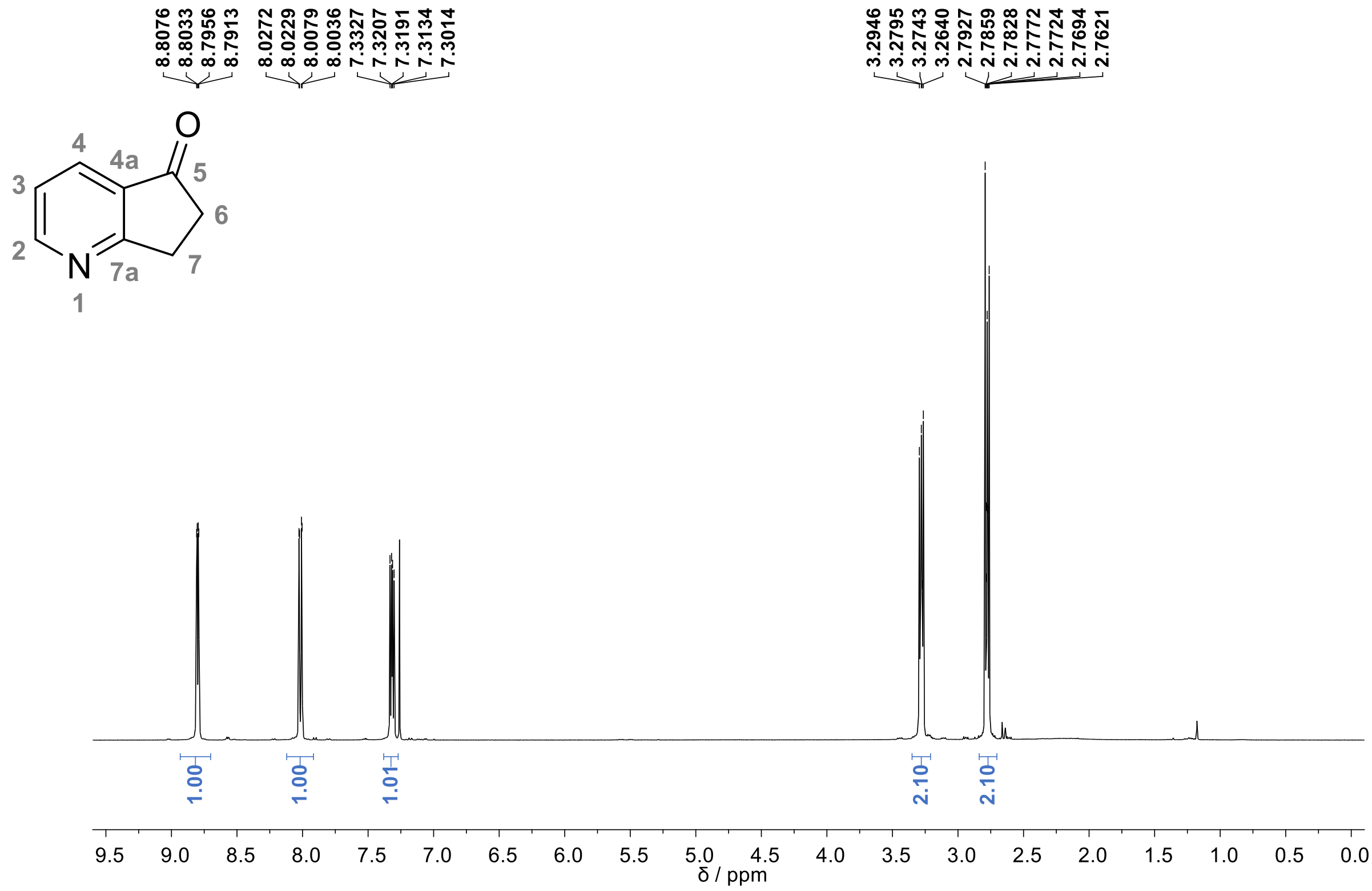

Figure S6. ^{1}H NMR spectrum ($CDCl_3$, 400 MHz) of ketone **6a**.

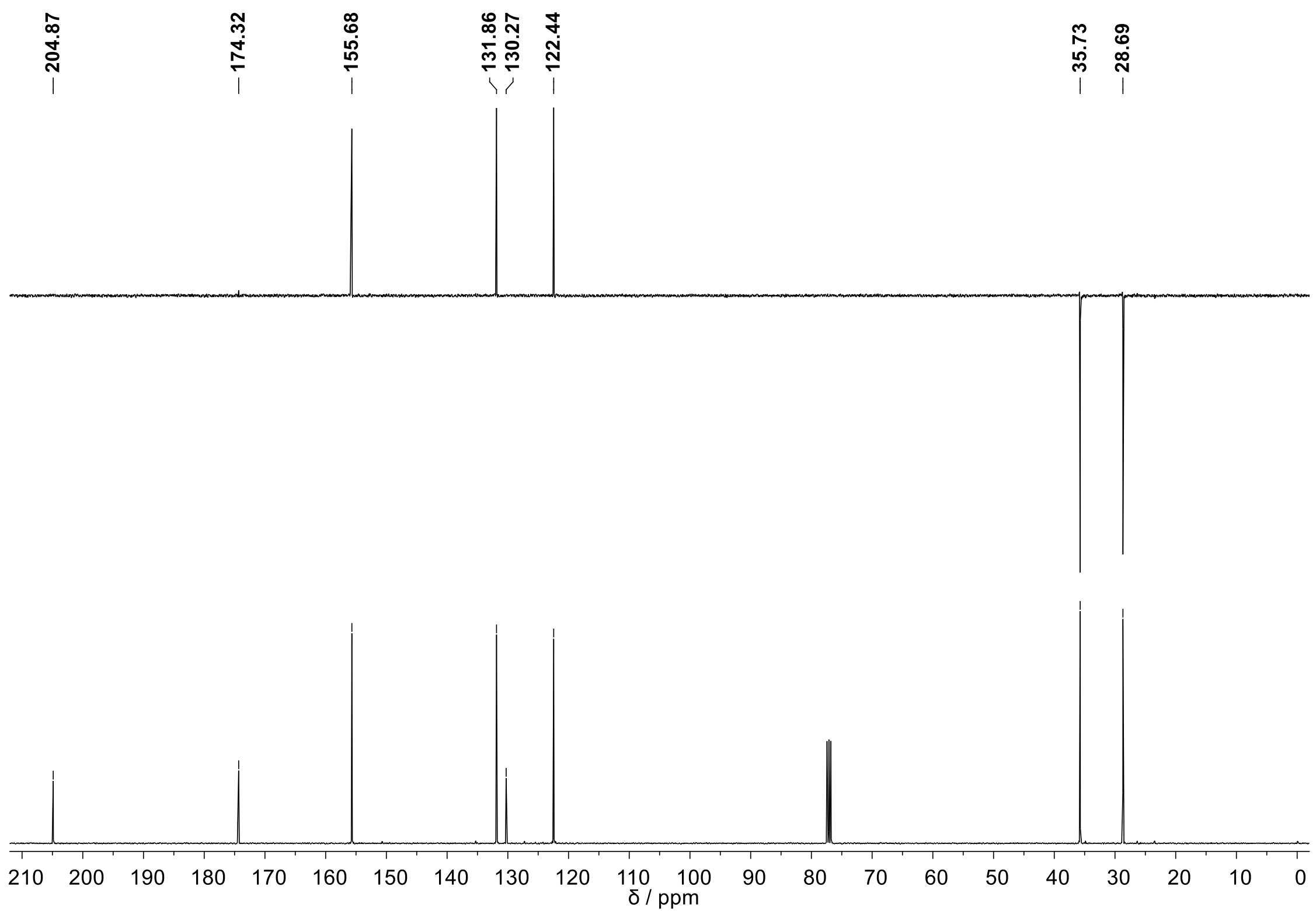

Figure S7. $^{13}C\{^{1}H\}$ and DEPT-135 NMR spectra ($CDCl_3$, 101 MHz) of ketone **6a**.

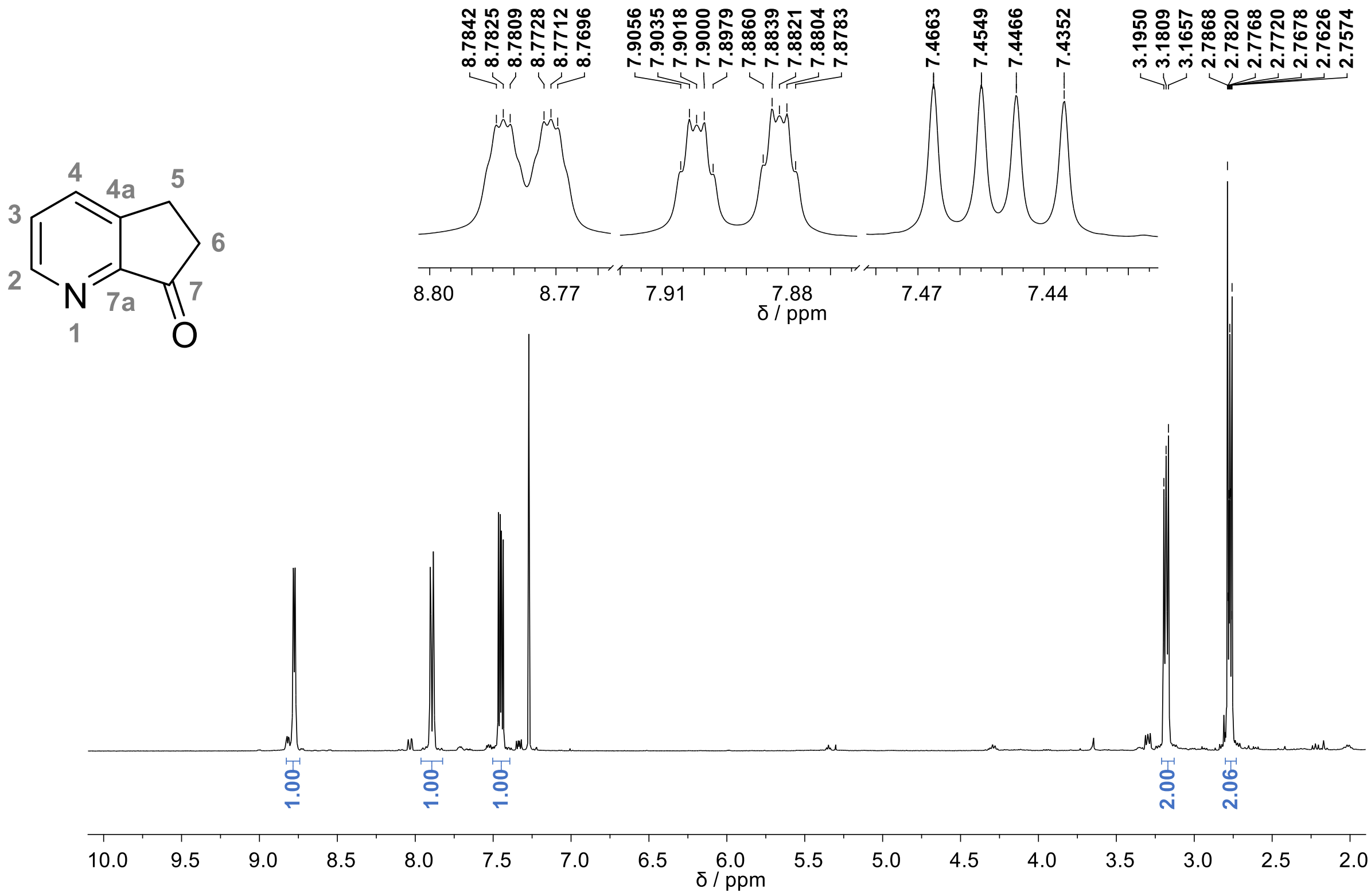

Figure S8. ^{1}H NMR spectrum ($CDCl_3$, 400 MHz) of ketone **6b**.

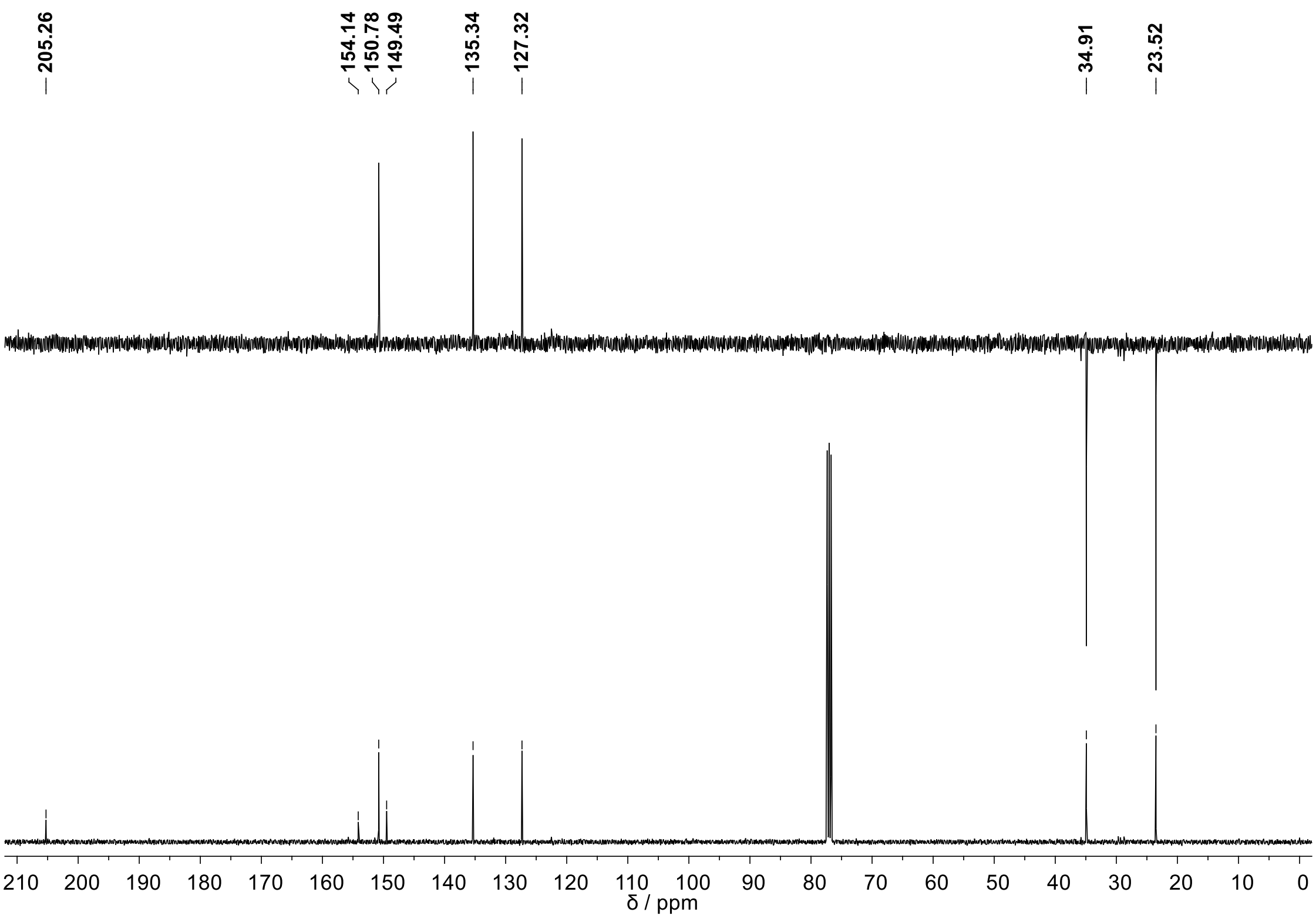

Figure S9. $^{13}C\{^{1}H\}$ and DEPT-135 NMR spectra ($CDCl_3$, 101 MHz) of ketone **6b**.

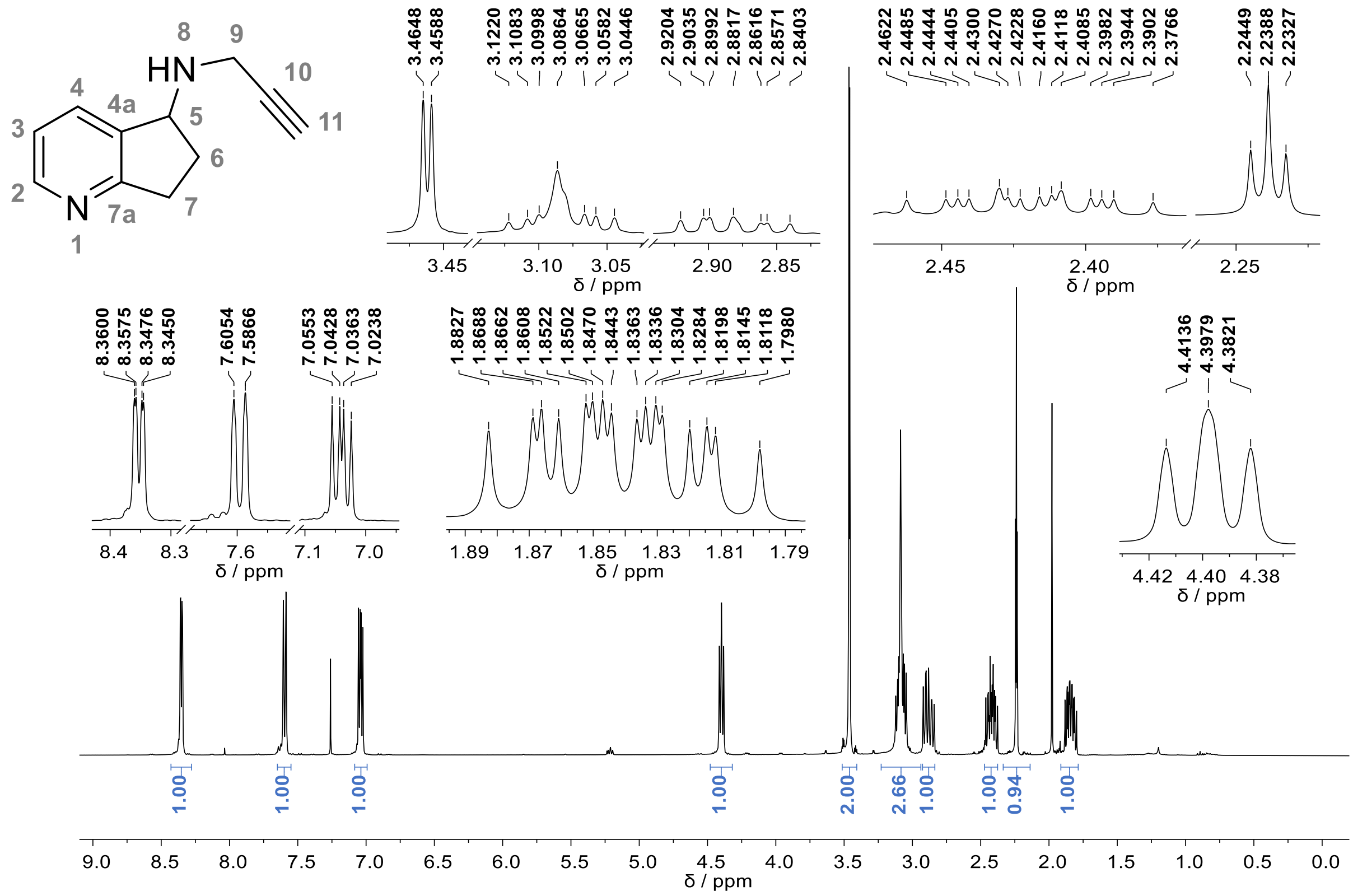

Figure S10. ^{1}H NMR spectrum ($CDCl_3$, 400 MHz) of *rac*-**2a**.

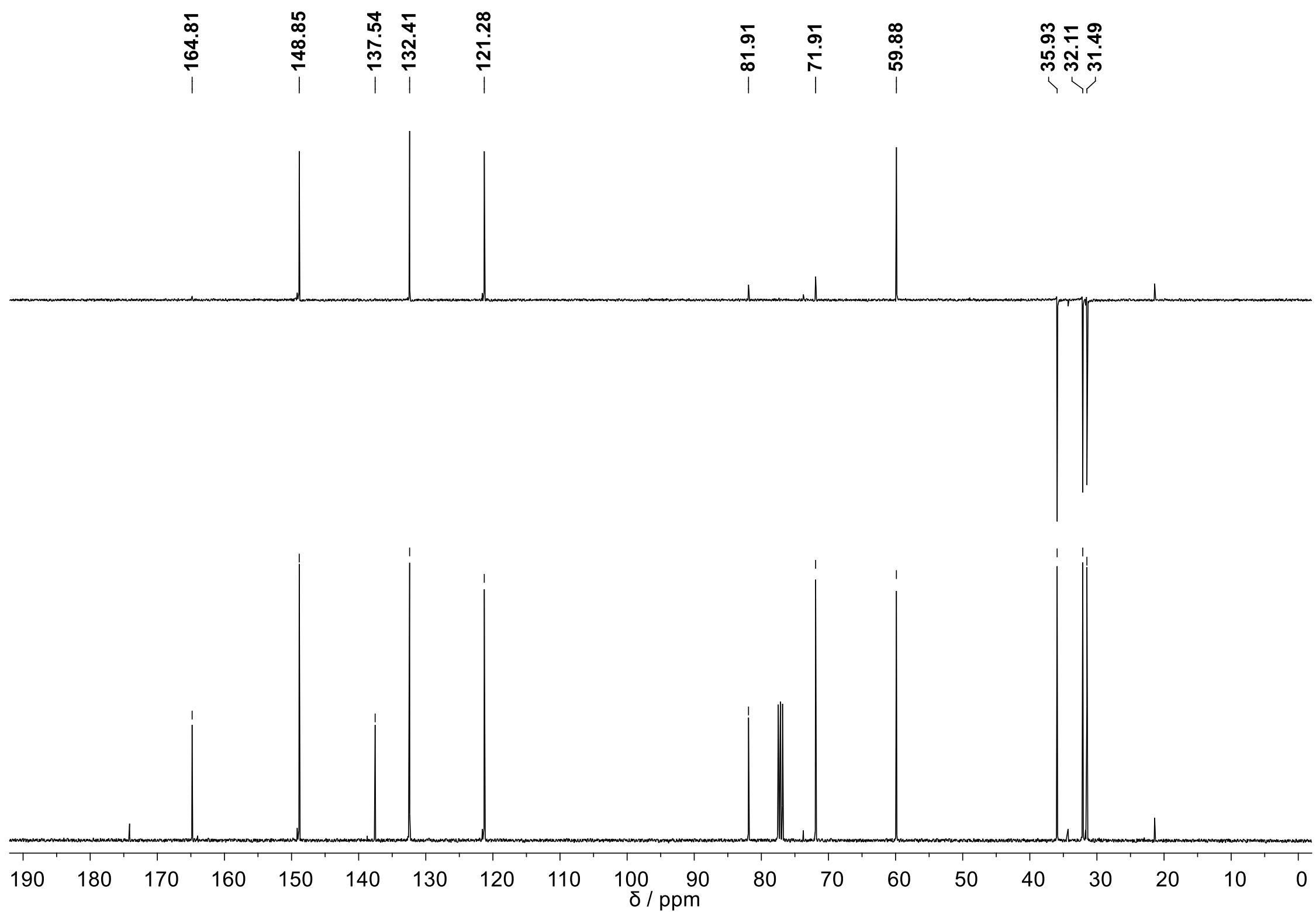

Figure S11. $^{13}C\{^{1}H\}$ and DEPT-135 NMR spectra ($CDCl_3$, 101 MHz) of *rac*-**2a**.

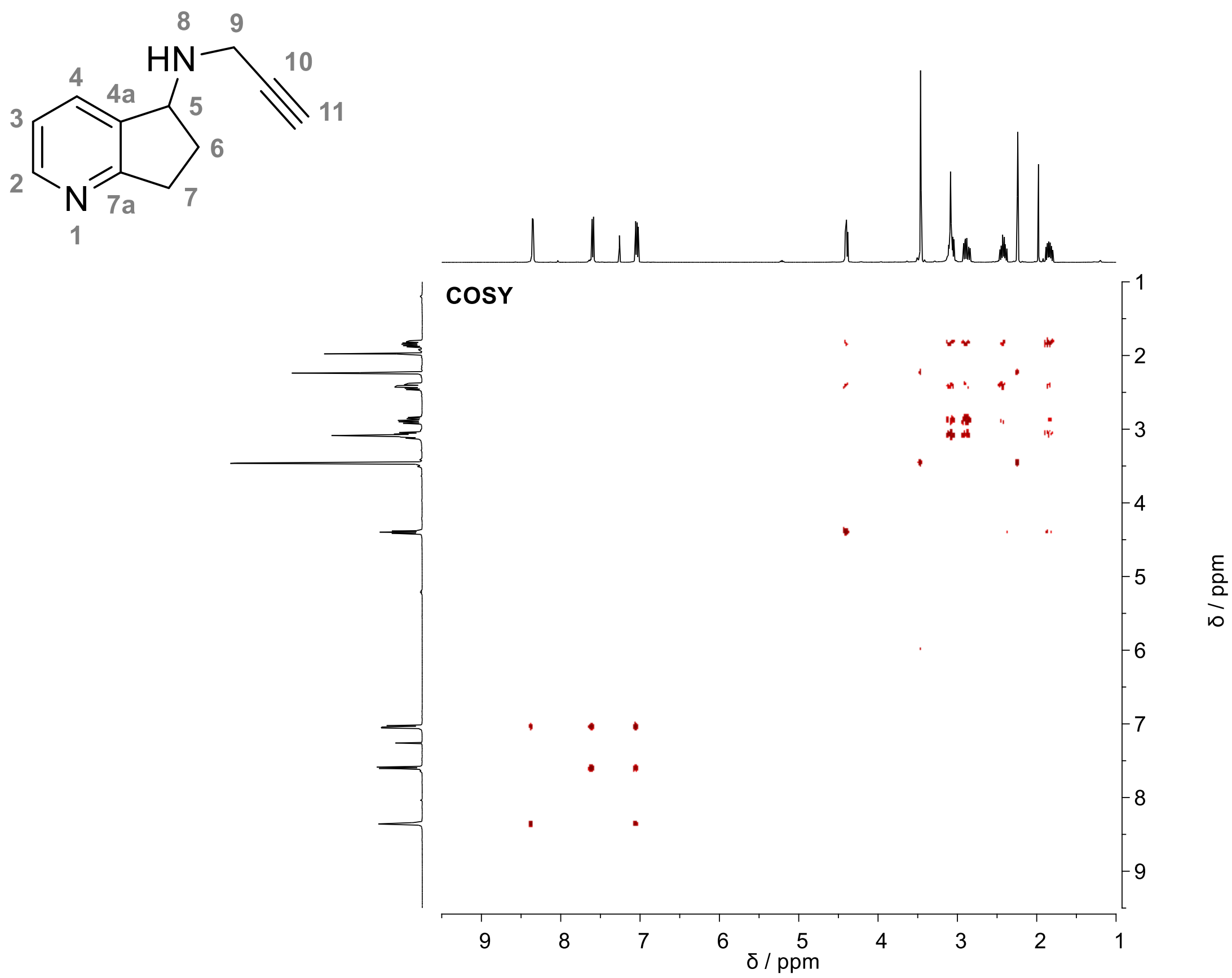

Figure S12. ^{1}H-^{1}H (COSY) spectrum ($CDCl_3$, 400 MHz) of *rac*-**2a**.

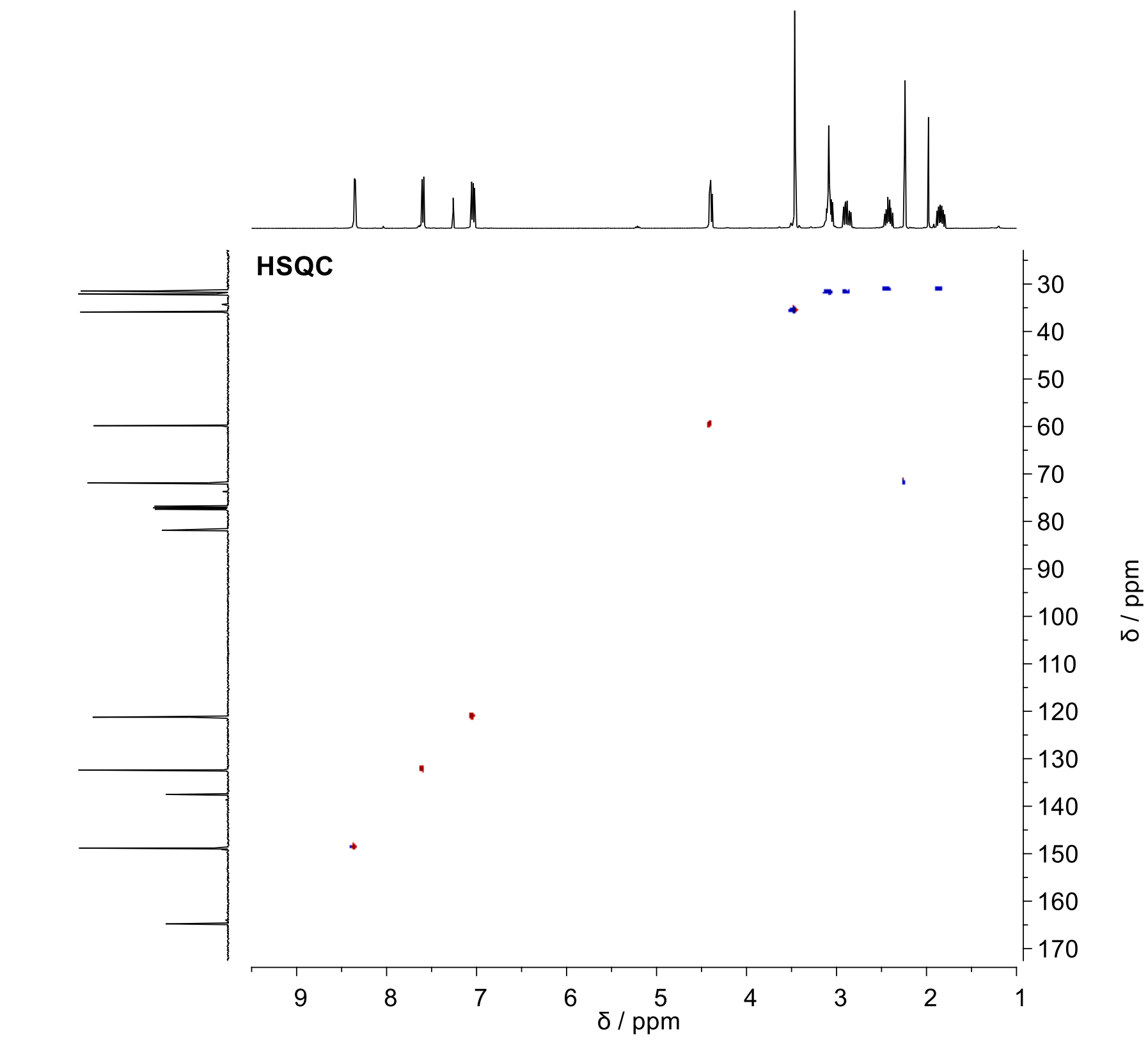

Figure S13. ^{1}H-^{13}C (HSQC) spectrum ($CDCl_3$, 400 MHz) of *rac*-**2a**.

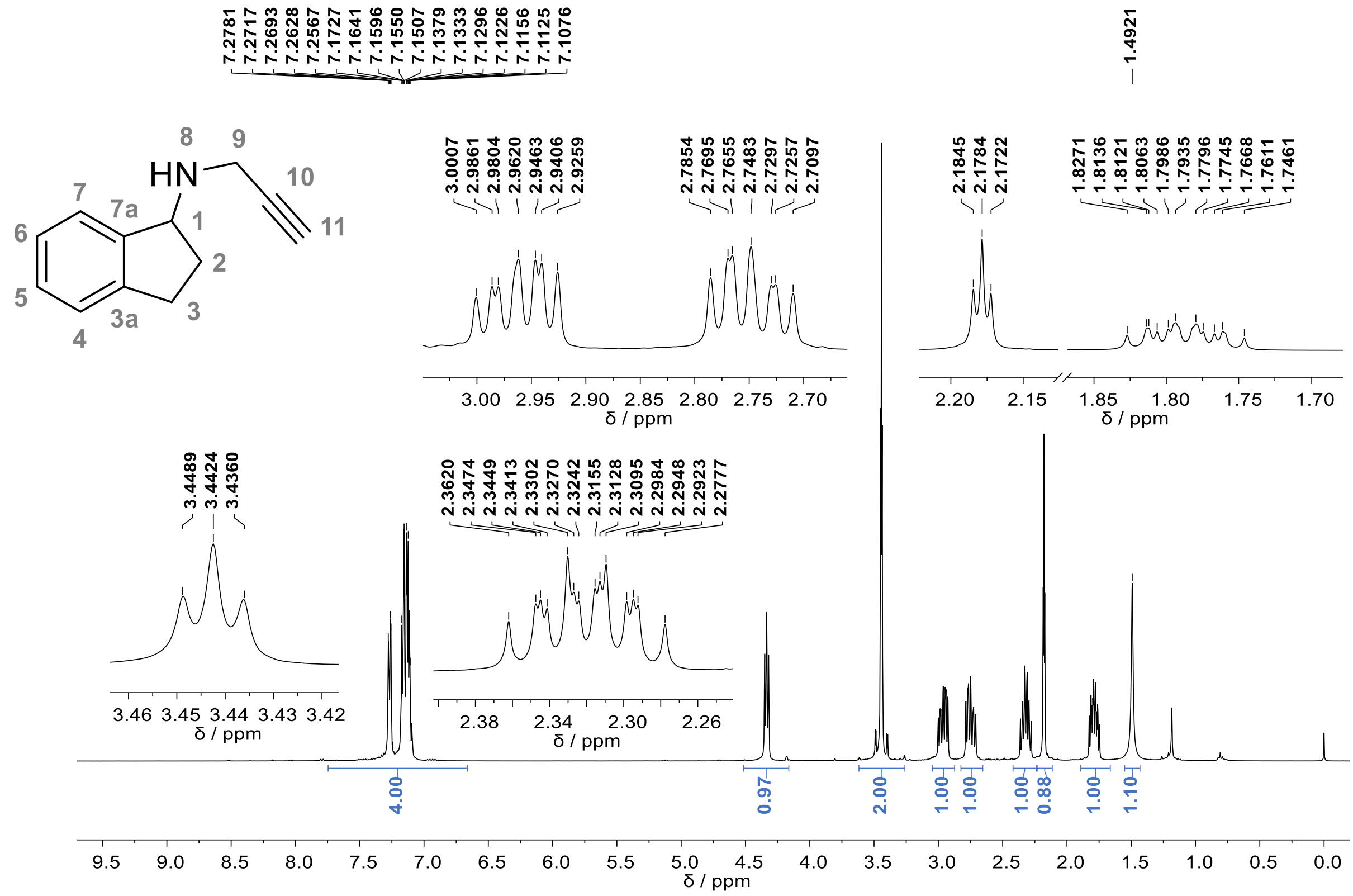

Figure S14. ^1H NMR spectrum ($CDCl_3$, 400 MHz) of *rac*-**1**.

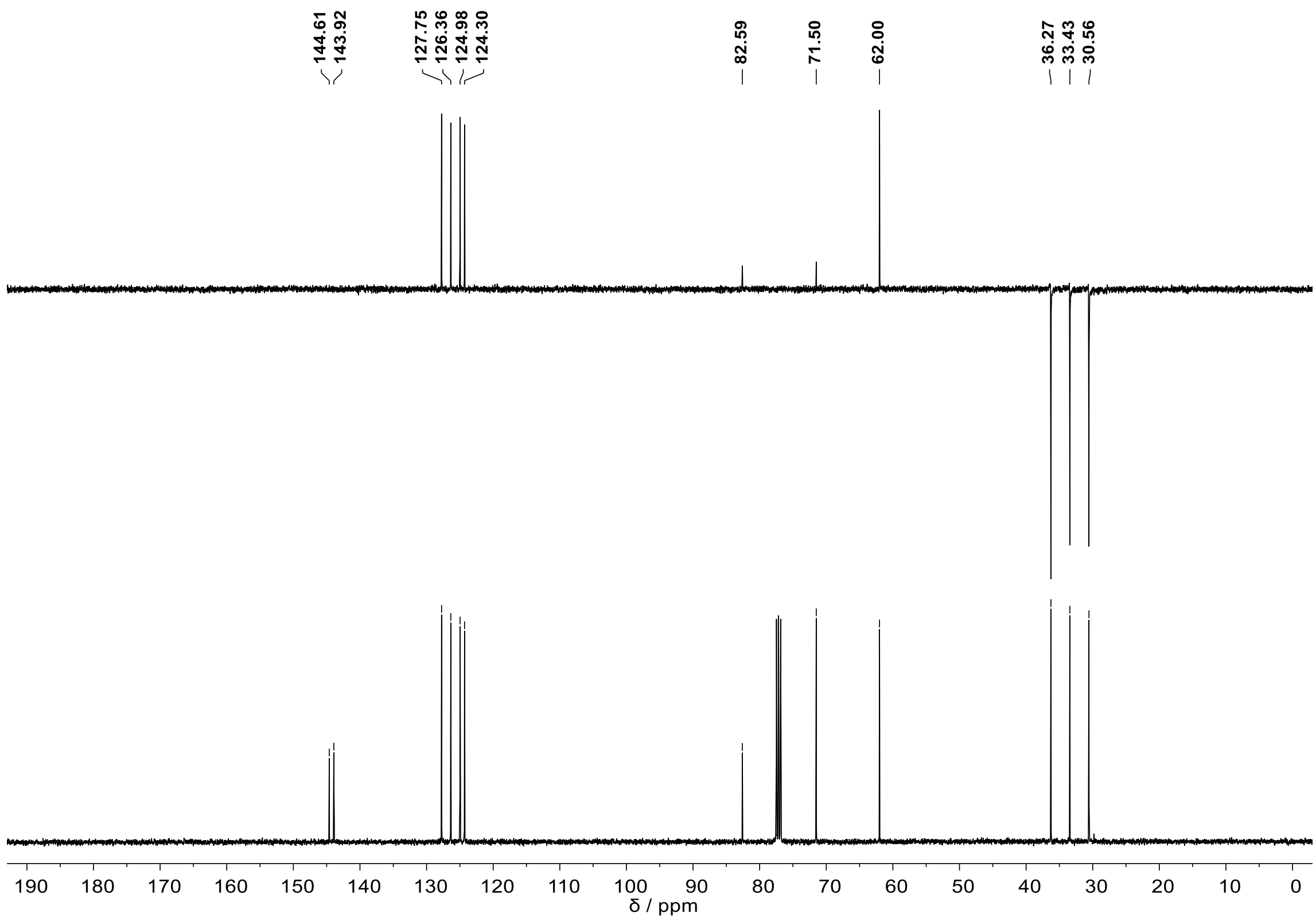

Figure S15. $^{13}C\{^1H\}$ and DEPT-135 NMR spectra ($CDCl_3$, 101 MHz) of *rac*-**1**.

4. HRMS Spectrum of *rac*-2a

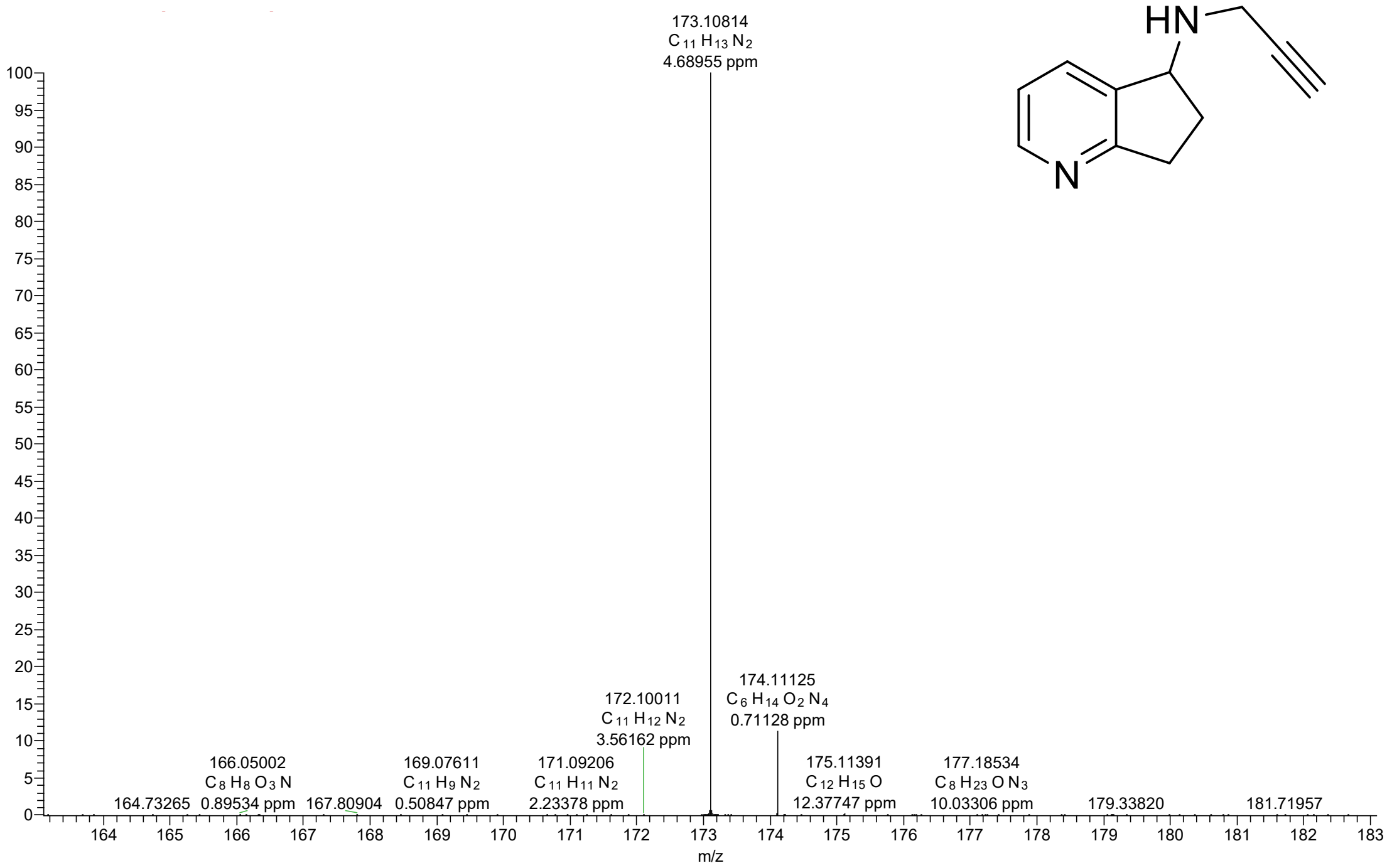

Figure S16. HRMS (ESI-TOF) *m/z* of *rac*-**2a**: $[M+H]^+$ calcd for $[C_{11}H_{13}N_2]^+$: 173.10732; obtained: 173.10814.

5. Toxicological Assays: Experimental Section

Cytotoxicity in Human Neuronal SH-SY5Y Cell Culture. To evaluate neurotoxicity, human neuroblastoma SH-SY5Y cells were used. These cells were obtained from Sigma-Aldrich (Taufkirchen, Germany). SH-SY5Y cells were maintained in a complete Dulbecco's Modified Eagle Medium (DMEM) [DMEM high glucose (D7777, Sigma-Aldrich, Germany)] supplemented with 10% (v/v) fetal bovine serum [Gibco-Invitrogen, from Alfagene (Carcavelos, Portugal)] and 1% (v/v) of antibiotics [100 units/mL penicillin and 100 µg/mL streptomycin (Biochrom, Berlin, Germany)] and maintained at 37 °C in an incubator with 5% CO_2 (Heraeus, Hanau, Germany). For experiments, cells were washed with phosphate-buffered saline (PBS) without calcium and magnesium (Biochrom, Berlin, Germany), trypsinized (trypsin/EDTA solution from Sigma-Aldrich, Germany), and counted using 0.4% (w/v) trypan blue solution (T8154, Sigma-Aldrich, Germany). The cell suspension was then seeded into multiwell plates at a density of 25,000 cells/cm^2 in complete DMEM containing 10 µM retinoic acid (R2625, Sigma-Aldrich, Germany) for 3 days. On day 3, cells were exposed to 80 nM phorbol 12-myristate 13-acetate (R79346, Sigma-Aldrich, Germany) in complete DMEM and kept for another 3 days to obtain a dopaminergic state. On day 6 of the differentiation protocol, new complete DMEM was given to the cells, and they were exposed to the compounds under study (**1**, *rac*-**1**, *rac*-**2a**, and *rac*-**2b** at 10 and 100 µM; and PQ at 300 µM) for 48 h.

All the compounds were solubilized in sterile PBS, except *rac*-**2b**, which was solubilized in sterile DMSO (Merck, Germany) (0.5% final well concentration). Thus, control cells were exposed to PBS in the same volume used for the compounds under study, while the vehicle of *rac*-**2b** was also tested in the same experimental paradigm as mentioned. The MTT reduction assay and NR uptake assay were used to determine the putative cytotoxicity of the drugs under study after the 48-h incubation.

Neuroprotection in Human Neuronal SH-SY5Y Cell Culture. Upon completion of the 6-day differentiation protocol, human dopaminergic SH-SY5Y cells were used to investigate the neuroprotective potential of **1**, *rac*-**1**, *rac*-**2a**, and *rac*-**2b** at 10 and 100 μM. A co-incubation strategy was adopted, whereby cells were pre-treated with each compound for 30 min before the neurotoxin PQ at 300 μM. Cell viability following the 48-h incubation period was assessed using the MTT reduction assay and NR uptake assay, as described below.

MTT Reduction Assay. In the colorimetric MTT assay, the tetrazolium salt is reduced to the corresponding formazan by dehydrogenases, mainly mitochondrial.[4] Briefly, after the 48-h incubation, the cellular medium was changed to new and warm complete DMEM with MTT (Alfa Aesar by Thermo Fisher Scientific, Kandel, Germany) (0.5 mg/mL final concentration). A 1.5 h incubation period at 37 °C followed (protected from light). The reaction was then stopped by removing the medium and adding DMSO to solubilize the formazan crystals, followed by a 15-min agitation period protected from light. The absorbance was read at 570 and 690 nm (reference wavelength) in a BioTek Synergy HT multi-well plate reader (Winooski, VT, USA), as described[5]. The values are expressed as a percentage of control cells that were set to 100%, after subtracting the absorbance reference value of each well. All conditions were performed in quadruplicate.

Neutral red (NR) uptake assay. At the end of the incubation time, the cell medium was removed, and 250 μL/well of NR solution (33 μg/mL of NR in DMEM) was added.[6] Then, plates were incubated for 1.5 h (at 37 °C, protected from light). After this time, the NR solution was removed, and 250 μL/well of HBSS was added for washing and then removed. To release the entrapped dye from viable cells, 100 μL/well of the solvent (50% ethanol/1% acetic acid) was added. The plates were then placed in a shaker for 30 min at room temperature, protected from light. Absorbance was measured at 540 and 690 nm in a BioTek Synergy HT multi-well plate reader (Winooski, VT, USA). All conditions were performed in quadruplicate.

6. *h*MAO Inhibition Assays

The effects of the test compounds on *h*MAO-A/B were investigated using a fluorimetric assay as previously described.[7] Recombinant *h*MAOs expressed in baculovirus-infected insect cells (BTI-TN-5B1-4), HRP (type II), and *p*-tyramine hydrochloride were purchased from Sigma-Aldrich. 10-Acetyl-3,7-dihydroxyphenoxazine (Amplex Red reagent) was synthesized according to the procedure described in the literature.[8]

Briefly, 100 µL of sodium phosphate buffer [50 mM, pH 7.4, 0.05% (v/v) Triton X-114] containing the compounds or the reference inhibitors and *h*MAO-A/B were incubated for 5, 15, or 30 min at 37 °C in a flat-bottomed 96-well black microplate. After the preincubation, the reaction was started by adding the final concentrations of 200 µM Amplex Red reagent, 1 U/mL HRP, and 1 mM *p*-tyramine (final volume, 200 µL). Resorufin production was quantified based on the fluorescence produced (λ_{ex} = 530 nm, λ_{em} = 590 nm) at 37 °C over a 30-min period. DMSO was used for the control experiments. For the determination of blank (*b*), the enzyme solution was replaced by a phosphate-buffered solution. Initial velocities were calculated from the trends obtained, with each measurement performed in duplicate. Inhibitory potencies are expressed as the residual activities, RA = $(v_i - b)/(v_o - b)$, where v_i is the velocity in the presence of the test compounds, and v_0 is the control velocity in the presence of DMSO. IC_{50} values were determined by plotting the residual enzyme activities against the applied inhibitor concentration, fitting the experimental data to a four-parameter Hill equation (Eq.1) using GraphPad Prism 9 (GraphPad Software, USA).

$$Y = \frac{\text{Bottom} + (\text{Top - Bottom})}{\left(1 + 10^{(\log IC_{50} - X) \times \text{Hill slope}}\right)} \qquad (Eq.\ 1)$$

7. References

1. Ren, L.; Wang, L.; Lv, Y.; Shang, S.; Chen, B.; Gao, S., Synthesis of 6,7-dihydro-5*H*-cyclopenta[*b*]pyridin-5-one analogues through manganese-catalyzed oxidation of the CH_2 adjacent to pyridine moiety in water. *Green Chem.* **2015**, *17* (4), 2369-2372.
2. Pereira, C. S.; Salgado, S.; Rizzo-Aguiar, F.; García-Mera, X.; Rodríguez-Borges, J. E., Synthesis of new propargylated 1-pyrindane derivatives as rasagiline analogues. *Synlett* **2013**, *24* (07), 837-838.
3. Rizzo-Aguiar, F.; Sousa, C. A. D.; García-Mera, X.; Rodríguez-Borges, J. E., Synthesis and characterization of 1-pyrindane derivatives as rasagiline analogues. *Chem. Data Collect.* **2016**, *5-6*, 21-27.
4. Dong, Y.; Berners-Price, S. J.; Thorburn, D. R.; Antatis, T.; Dickinson, J.; Hurst, T.; Qiu, L.; Khoo, S. K.; Parsons, P. G., Serine protease inhibition and mitochondrial dysfunction associated with cisplatin resistance in human tumor cell lines: Targets for therapy. *Biochem. Pharmacol.* **1997**, *53* (11), 1673-1682.
5. Sampaio-Dias, I. E.; Reis-Mendes, A.; Costa, V. M.; García-Mera, X.; Brea, J.; Loza, M. I.; Pires-Lima, B. L.; Alcoholado, C.; Algarra, M.; Rodríguez-Borges, J. E., Discovery of new potent positive allosteric modulators of dopamine D_2 receptors: Insights into the bioisosteric replacement of proline to 3-furoic acid in the melanostatin neuropeptide. *J. Med. Chem.* **2021**, *64* (9), 6209-6220.
6. Dionísio, F.; Araújo, A. M.; Duarte-Araújo, M.; Bastos, M. L.; Guedes de Pinho, P.; Carvalho, F.; Costa, V. M., Cardiotoxicity of cyclophosphamide's metabolites: An in vitro metabolomics approach in AC16 human cardiomyocytes. *Arch. Toxicol.* **2022**, *96* (2), 653-671.
7. Knez, D.; Colettis, N.; Iacovino, L. G.; Sova, M.; Pišlar, A.; Konc, J.; Lešnik, S.; Higgs, J.; Kamecki, F.; Mangialavori, I.; Dolšak, A.; Žakelj, S.; Trontelj, J.; Kos, J.; Binda, C.; Marder, M.; Gobec, S., Stereoselective activity of 1-propargyl-4-styrylpiperidine-like analogues that can discriminate between monoamine oxidase isoforms A and B. *J. Med. Chem.* **2020,** *63* (3), 1361-1387.
8. Von der Eltz, H.; Guder, H.-J.; Muhlegger, K. New hydrolase substrates. U.S. Patent 4,900,822, February 13, 1990.
